# Microscopic Visualization of Cell-Cell Adhesion Complexes at Micro and Nanoscale

**DOI:** 10.3389/fcell.2022.819534

**Published:** 2022-04-20

**Authors:** Bieke Vanslembrouck, Jian-hua Chen, Carolyn Larabell, Jolanda van Hengel

**Affiliations:** ^1^ Molecular Biophysics and Integrated Bioimaging, Lawrence Berkeley National Laboratory, Berkeley, CA, United States; ^2^ Department of Anatomy, University of San Francisco, San Francisco, CA, United States; ^3^ Medical Cell Biology Research Group, Department of Human Structure and Repair, Faculty of Medicine and Health Sciences, Ghent University, Ghent, Belgium

**Keywords:** cell-cell adhesion, adherens junction, desmosome, tight junction, intercellular junctions, imaging techniques, microscopy, electron microscopy

## Abstract

Considerable progress has been made in our knowledge of the morphological and functional varieties of anchoring junctions. Cell-cell adhesion contacts consist of discrete junctional structures responsible for the mechanical coupling of cytoskeletons and allow the transmission of mechanical signals across the cell collective. The three main adhesion complexes are adherens junctions, tight junctions, and desmosomes. Microscopy has played a fundamental role in understanding these adhesion complexes on different levels in both physiological and pathological conditions. In this review, we discuss the main light and electron microscopy techniques used to unravel the structure and composition of the three cell-cell contacts in epithelial and endothelial cells. It functions as a guide to pick the appropriate imaging technique(s) for the adhesion complexes of interest. We also point out the latest techniques that have emerged. At the end, we discuss the problems investigators encounter during their cell-cell adhesion research using microscopic techniques.

## 1 Introduction

Proper adhesion between cells is critical for the biogenesis and maintenance of many tissue types and disrupted adhesion is commonly seen in many disorders, including carcinomas (Salvador et al., 2016), asthma (Wittekindt 2017), and inflammatory bowel diseases (Lee 2015). Cell-cell adhesion is regulated by three major junctional complexes: desmosomes, adherens junctions, and tight junctions (also called macula adherens, zonula adherens and zonula occludens, respectively). Desmosomes and adherens junctions are mainly responsible for strong adhesion between cells, while tight junctions control the paracellular permeability as diffusion barriers. Tight junctions are also thought to play a crucial role in controlling the epithelial cell-polarization forming a border between the apical and basolateral cell surface domains (Zihni et al., 2016; Otani and Furuse, 2020). Each comprises a wide range of proteins that drive junctional assembly and dynamics but also the mechanical coupling between cells; their expression and activity must therefore be precisely regulated in order to maintain proper homeostasis. Another form of intercellular coupling is facilitated by gap junctions; they provide the electrical coupling and currents at cell-cell contacts. There are some reviews about functional gap junction coupling (Nielsen et al., 2012; Stephan et al., 2021). In this review, we focus on mechanical coupling. We summarize the imaging techniques used to study the structure and composition of the three major adhesion complexes (desmosomes, adherens junctions, and tight junctions) from epithelial and endothelial tissue. In addition, we give examples of experiments in which microscopy techniques have been used to answer questions in the field, focusing on both the cell-cell connection and the connection with the cytoskeleton.

### 1.1 The Main Intercellular Junctions and Their Proteins

**FIGURE 1:**
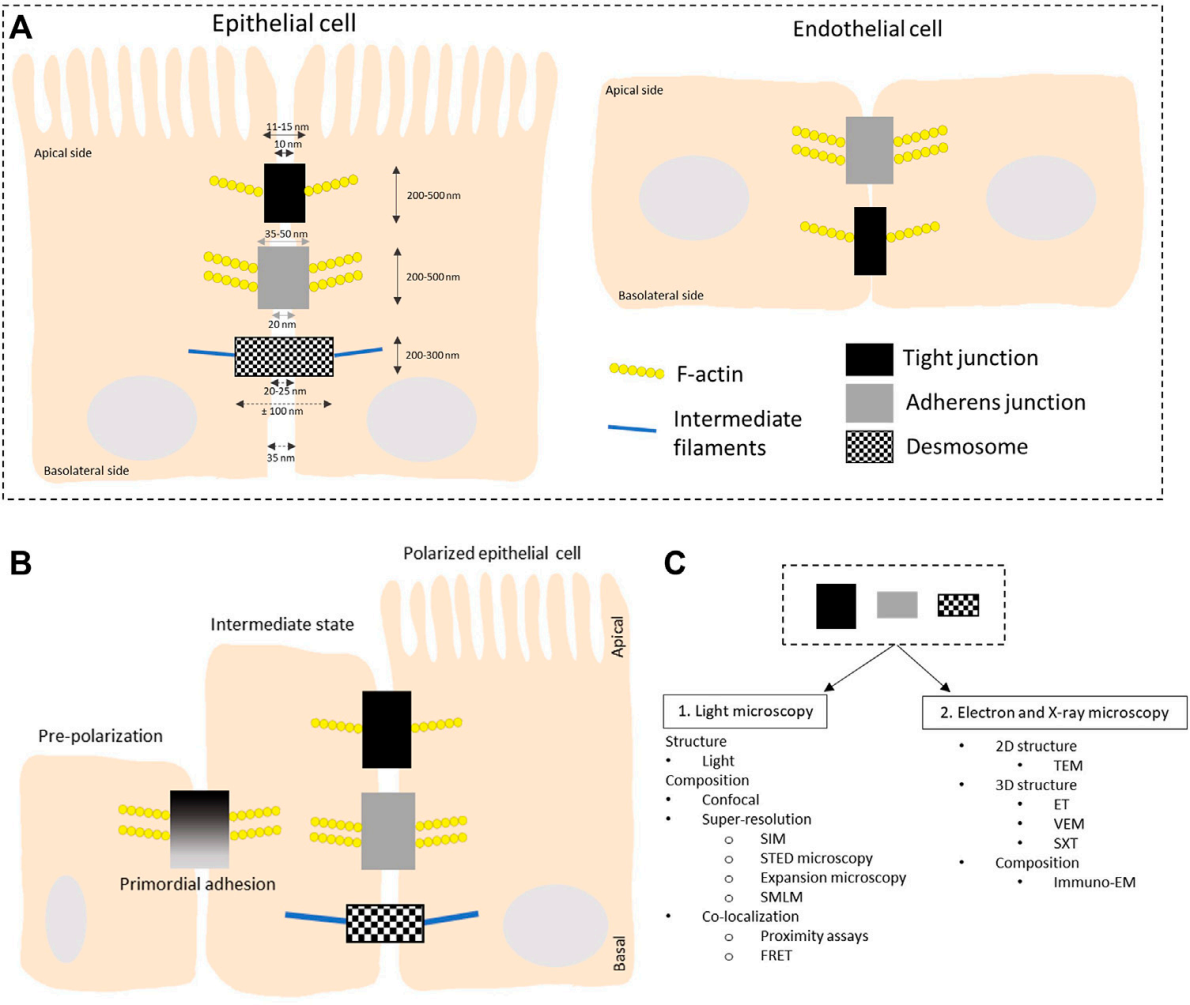
Schematic overview of the organization of intercellular junctions in epithelial and endothelial cells. **(A)** Tight junctions, adherens junctions and desmosomes are present in polarized epithelial cells while no desmosomes are found in flat endothelial cells. Tight and adherens junctions are connected to the actin cytoskeleton while desmosomes connect to intermediate filaments. **(B)** Organization of intercellular junctions during the polarization of epithelial cells. In the initial stage of polarization, proteins like afadin and nectins are present. They recruit E-cadherin and junctional adhesion molecules (JAMs) to the lateral cell-cell contacts, called the primordial adhesions in immature epithelial cells. These mature to form belt-like adhesion junctions and tight junctions localized at the apical-basal membrane border. The mechanism behind the polarization is reviewed in (Martin-Belmonte and Perez-Moreno, 2012). The overall height of a polarized cell grown *in vitro* is ∼ 15 μm, figures are not on scale. **(C)** Overview of the different microscopy techniques that are discussed in this review to study the structure and composition of the three types of intercellular junctions. LM: light microscopy; TEM: transmission electron microscopy; ET: electron tomography; VEM: volume electron microscopy; SXT: soft X-ray microscopy; SIM: structured illumination microscopy; STED: stimulated emission depletion; SMLM: single molecule localization microscopy; FRET: Forster resonance energy transfer; Immuno-EM: immuno electron microscopy.

**FIGURE 2:**
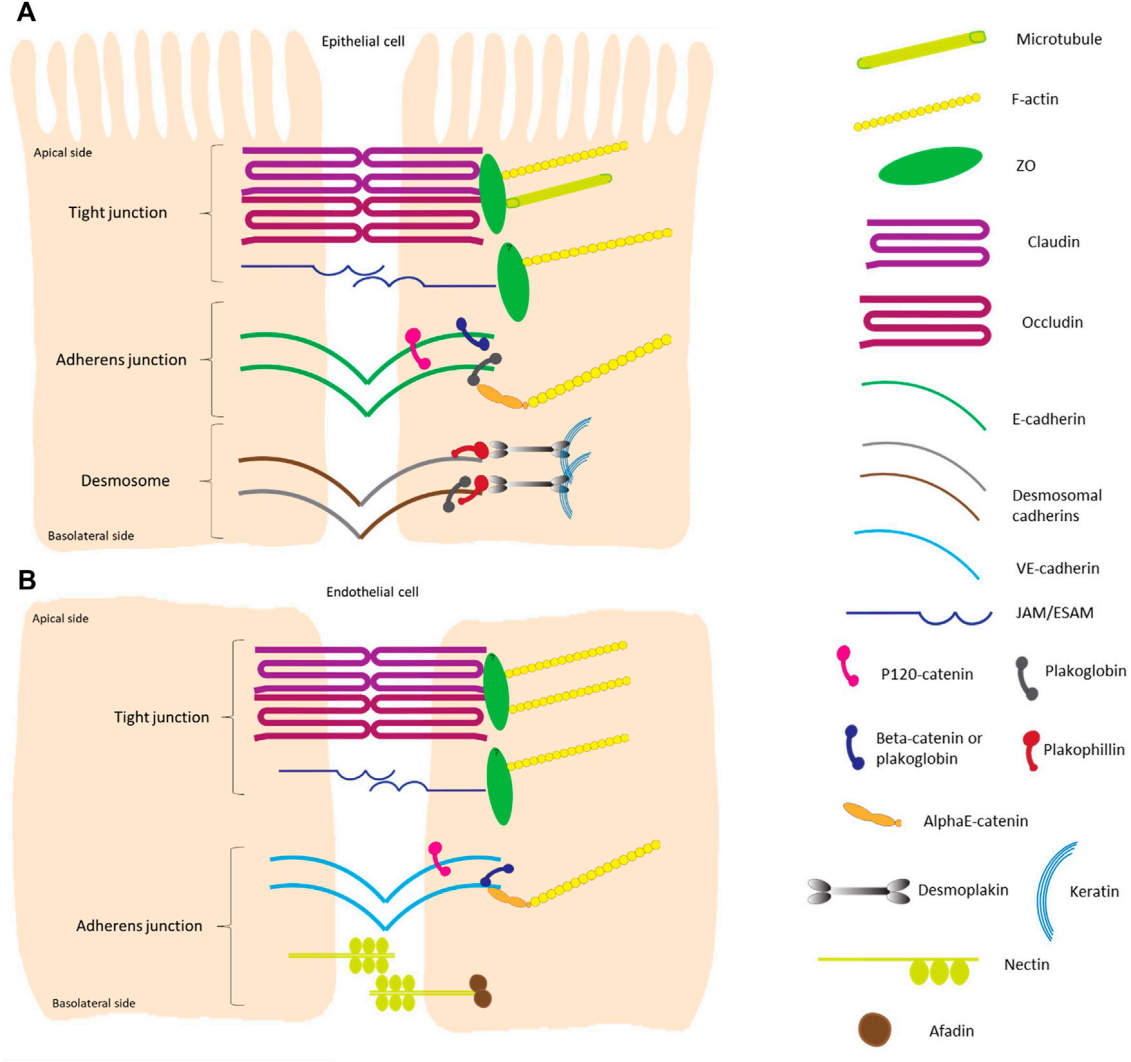
Schematic overview of the different components of intercellular junctions in epithelial and endothelial cells. **(A)** Highly polarized epithelial cell-cell contacts are composed of tight junctions, adherens junctions and desmosomes from the apical to basal side. The transmembrane proteins of tight junctions are claudins, which largely determine the paracellular ion permeability between cells, and proteins like occludin and junctional adhesion molecules (JAMs) (Zihni et al., 2016; Otani and Furuse, 2020). The cytosolic plaque of the tight junctions is a complex protein network, with adaptor proteins containing multiple protein-protein interaction motifs that are connected to F-actin and microtubules. One of the main cytoplasmic tight junction proteins are of the zonula occludens (ZO) family. Adherens junctions consist of transmembrane cadherin proteins, such as E-cadherin, and cytoplasmic proteins that are members of the catenin family; p120-catenin binds to E-cadherin closest to the intercellular space while α-catenins anchor the cadherin-catenin complex to the actin cytoskeleton by binding to the armadillo proteins β-catenin and/or plakoglobin (Gumbiner 2005; Perez-Moreno and Fuchs, 2006; Meng and Takeichi, 2009). The main transmembrane proteins in the desmosomal complex are two types of cadherins: desmogleins and desmocollins. They form heterodimers that make up the fundamental adhesive unit of desmosomes (Harrison et al., 2011). The cytoplasmic side of the desmosomal cadherins are linked with plakophilin and plakoglobin, which in their turn bind both to desmoplakin to make up the connection to the intermediate filament (keratin) complex of the cell (Green and Gaudry, 2000; Garrod and Chidgey, 2008). **(B)** Tight junctions in flat endothelial cells are very similar to those in polarized epithelial cells. The major difference is that there is no microtubule to bind to in the endothelial cells. Adherens junctions consist of vascular endothelial cadherin (vascular-endothelial) in endothelial cells and do not contain plakoglobin as cytoplasmic protein. Transmembrane nectin protein attached to the cytoplasmic afadin can be found in endothelial cells (Wallez and Huber, 2008). The overall height of a polarized cell grown *in vitro* is ∼15 μm, figures are not on scale.

Tight junctions, adherens junctions and desmosomes are composed of transmembrane proteins that form extracellular adhesive contacts between cells while intracellularly, the junctional proteins are also linked with the cytoskeletal structural components of the cell (Hartsock and Nelson, 2008). Both adherens and tight junctions are closely associated with a circumferential belt of actin filaments. In this review we cover imaging techniques used to study the adhesion complexes in highly polarized epithelial cells and flat endothelial cells (Figure 1). Proteins in the tight junction barrier are known to regulate intercellular communication and paracellular transport between cells. These tight junctions between two neighboring cells are often 200–500 nm in length and 11–15 nm wide, while the intermembrane space at these junctions is only 10 nm (Farquhar and Palade, 1963). Adherens junctions are important for multiple functions including initiation and stabilization of cell-cell adhesion, regulation of actin cytoskeleton, intracellular signaling and transcriptional regulation. These junctions are 200–500 nm long and 35–50 nm wide with 20 nm gap between opposing membranes. Desmosomes provide strong adhesion between cells and mediate cell-cell contact. Desmosomes are 200–300 nm long and can span up to 100 nm in width while the intermembrane space varies between 20 and 25 nm. Reviewed in Adil et al. (2021) and more references are found in the legend of Figure 2.

These spatially defined adhesion complexes are also known as signaling hubs that cross-talk in order to coordinate tissue organization and function. Malfunction of one type of adhesion complex, for instance by deleting one of the crucial junctional proteins, not only affects the function and/or organization of that specific junction type but can also impair other intercellular junctions [reviewed in (Rübsam et al., 2018)]. Moreover, in some tissue types mixed types of junctions can be found, i.e., area compositae at the intercalated disc of cardiomyocytes. There desmosomal proteins can be found in the adherens junction area (Borrmann et al., 2006; Franke et al., 2006). In flat endothelial cells, tight junctions and adherens junctions can overlap and intercalate. Tight junctions in endothelial cells are very similar to the ones in polarized epithelial cells, with the major difference that there is no microtubule to bind to in the endothelial cells (Figures 1, 2).

In addition to junctions between two neighboring cells, tricellular junctions can be formed at the corners where three cells meet. Consequently, tricellular contacts require more complex junctions and are reflected in the components present, such as angulins and tricellulin in the tight junctions at these tricellular borders (Zihni et al., 2016) or another plakophilin (Pkp) isoform in tricellular junctions in keratinocytes (Keil et al., 2016; Rietscher et al., 2018).

### 1.2 Visualizing the Junction With Microscopic Imaging

Microscopy is a fundamental part of current research and is used by researchers to understand the mechanisms of human health and diseases on the cellular level. Different imaging purposes can be served depending on the research question. This can be the investigation of specific proteins, the study of the ultrastructure of tissue and the behavior of certain complexes in diseased states. Broadly, when studying intercellular junctions, microscopy can be used to either look at the structure, the composition, or the activity of the adhesions. When looking at the (ultra)structure and composition of cells, several organelles and cellular entities are visible on a micro- or nanoscale, depending on the resolution of the microscopic technique used.

**FIGURE 3:**
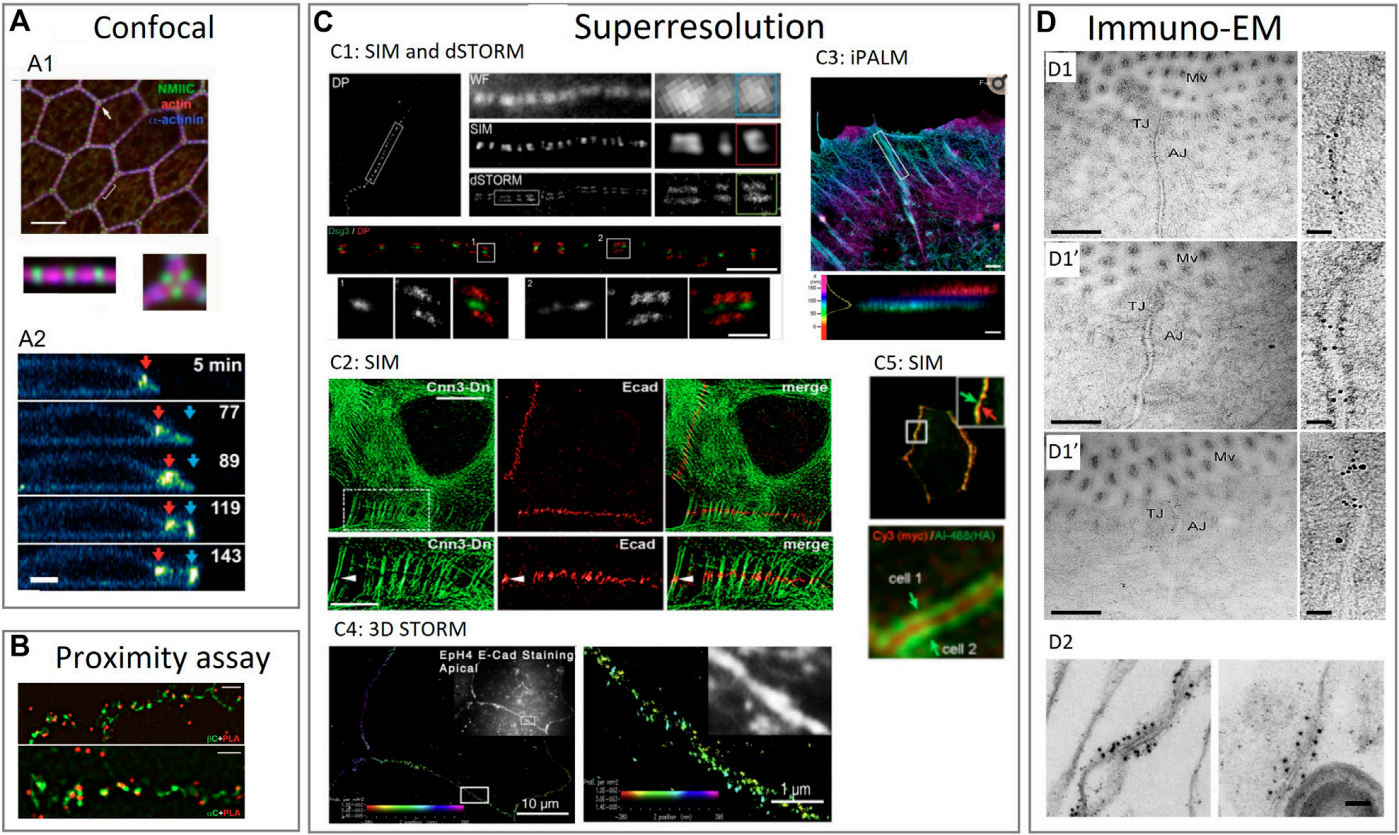
Examples of using various imaging techniques to study the composition of intercellular junctions. **(A)** Confocal imaging techniques to study the composition of tight junctions, adherens junctions and desmosomes. **(A1)**: Confocal imaging of apical cell junctions and the actin network around it. Actin and α-actinin1 co-localization and the alternation with non-muscle myosin II-C (NMIIC). Fluorescent intensity (FI) profile of NMIIC, actin and α-actinin was presented (Ebrahim et al., 2013). **(A2)**: Confocal imaging of desmosomes during apoptosis. Transverse view of a neighboring cell showing desmosomal junction dynamics during apoptotic cell extrusion. Red and blue arrows indicate pre-existing and *de novo* desmosomal junctions (Thomas et al., 2020). **(B)** Proximity assay techniques to study the composition of tight junctions, adherens junctions and desmosomes. Proximity ligation assay (PLA) in epithelial cells to determine the localization of adherens junction proteins. Cells stained with anti-β-catenin or anti-α-catenin mouse antibodies were imaged by structured illumination microscopy (SIM) super-resolution microscopy (Indra et al., 2013). **(C)** Superresolution imaging techniques to study the composition of tight junctions, adherens junctions and desmosomes. **(C1)**: SIM and dSTORM imaging of desmoplakin and desmoglein proteins in desmosomes. Upper part: SIM image of desmoplakin cell border. Region also imaged with WF (wide field) microscopy, SIM and dSTORM. Bottom part: dSTORM image of keratinocyte cell-cell border labeled for the desmoglein-3 (Dsg3) N terminal (green) and desmoplakin C-terminal (red) domains (Stahley S. N. et al., 2016). **(C2)**: Localization of E-cadherin with actin visible by SIM. SIM of A431 cells expressing actin binding protein (calponin3) and double-stained for E-cadherin to show locations of punctate adherens junctions (Indra et al., 2020). **(C3)**: interferometric photoactivated localization microscopy **(**iPALM) imaging of F-actin in epithelials cells. F-actin is labeled with phalloidin. Colors indicate the vertical (z) coordinate, relative to the substrate surface (Bertocchi et al., 2017). **(C4)**: 3D STORM image of apical cell-cell junctions between Eph4 cells shows E-cadherin staining. In all 3D-STORM images, the z position is color coded, and intensity indicates position accuracy according to the color bar in each panel (Wu et al., 2015). **(C5)**: Cells expressing exogenous ZO-1 labeled on the N-terminus and on the C-terminus made visible by SIM. Upper panel: exogenous ZO-1 targeted to junctions. Lower panel: central N-terminal (red) labeled region flanked by two C-terminal (green) labeled regions of ZO-1, corresponding to a mirror-like arrangement of ZO-1 molecules on two sides (Spadaro et al., 2017). **(D)** Immuno-electron microscopy imaging techniques to study the composition of tight junctions, adherens junctions and desmosomes. **(D1)**: Immuno electron microscopy (immuno-EM) images double labeled with immunogold for different tight and adherens junction proteins. Enterocyte tissue of normal mice with occludin labelled with 10 nm gold which localizes mainly at the tight junctions **(D1)**, calveolin-1 labelled with 10 nm gold which localizes mainly at the adherens junctions **(D1’)** and occludin labelled with 10 nm gold and calveolin with 15 nm gold to see the latter in both the adherens and tight junctions while occluding only occurs at the tight junction **(D1’’)** (Marchiando et al., 2010). **(D2)**: Immuno-EM of isolated chick liver tissue to show the tight junction protein occluding labelled with 10 nm gold particle. The tight junction strand is cut traverse and many gold particles are aligned longitudinal to the cut (Furuse et al., 1993). Reproduced/adapted with permission.

**FIGURE 4:**
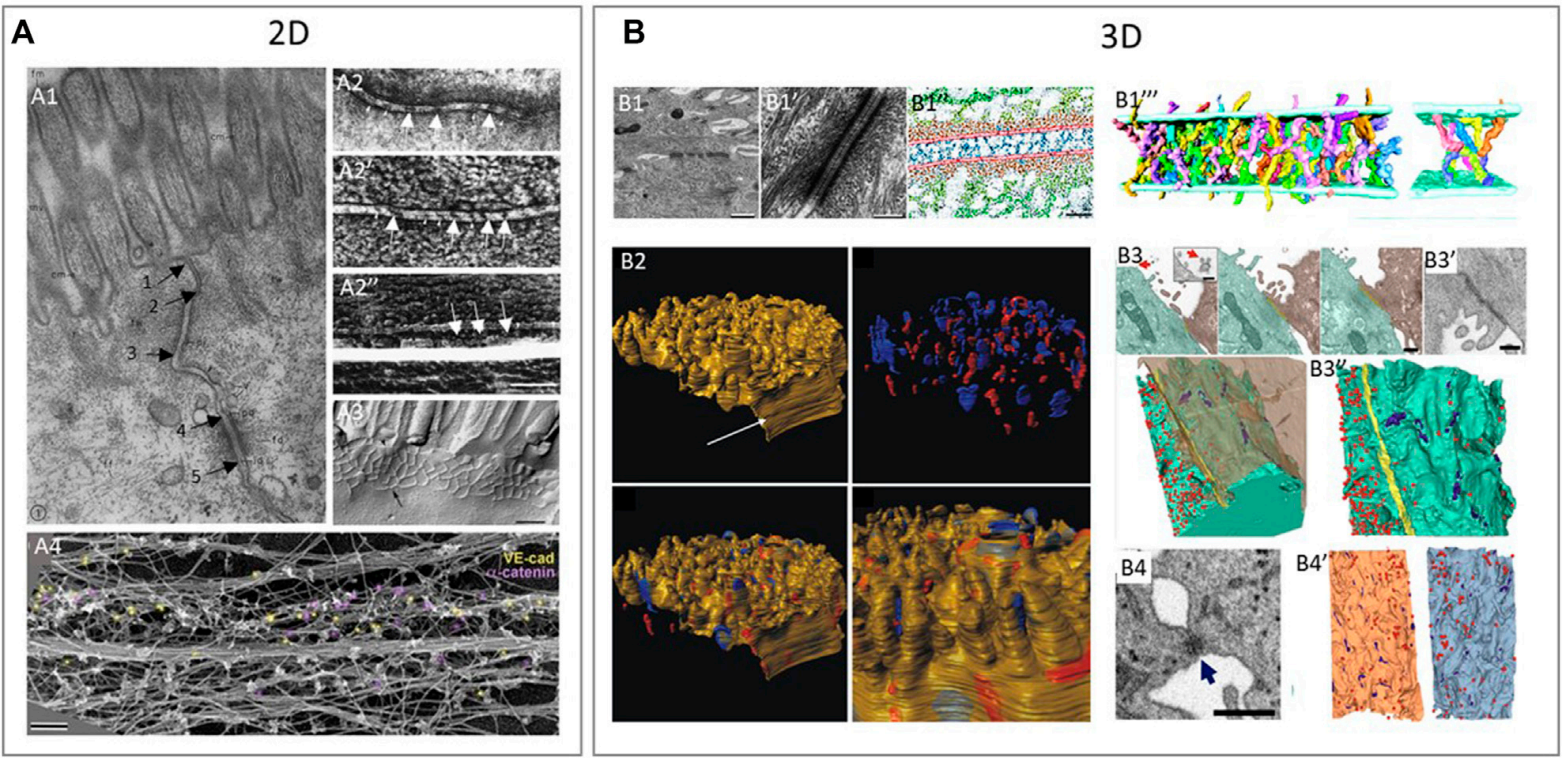
Examples of using various 2D and 3D imaging techniques to study the structure of intercellular junctions. **(A)** 2D imaging techniques to study the structure of tight junctions, adherens junctions and desmosomes. **(A1)**: Transmission electron microscopy (TEM) shows a tight junction (arrow 1–2), adherens junction (arrow 2–3) and desmosome (arrow 4–5) in epithelium of intestinal mucosa of rat (Farquhar and Palade, 1963). **(A2)**: Freeze fractured EM (FFEM) of epithelial junctions in which adherens junction is seen as a dense central structure between membranes **(A2,A2’)** and the underlying cytoplasmic actin bundles **(A2’’)** in retinal pigment epithelium (Miyaguchi 2000). **(A3)**: FFEM of tight junctions in mouse epithelial cells (Furuse 2010) A4: Platinum replica electron microscopy (PREM) is an EM based technique that allows researchers to image actin bundles next to junctions and in this study, researchers showed vascular-endothelial-cadherin (yellow) and α-catenin (purple) by immunogold labelling in relation to the actin cytoskeleton of linear endothelial adherens juctions (Efimova and Svitkina, 2018). **(B)** 3D techniques to study the structure of tight junctions, adherens junctions and desmosomes. **(B1)**: Desmosomes between keratinocytes in neonatal mouse epidermis imaged by electron tomography (ET). ET image of desmosome **(B1-B1’)** and the cadherin molecules crossing the extracellular space (blue in B1’’) in an annotated image. Reconstruction of individual cadherin molecules of a desmosome **(B1’’’)** (He et al., 2003). **(B2)**: 3D representation of a murine cardiac intercalated disc imaged by focused ion beam scanning EM (FIB-SEM). Desmosomes (blue) and gap junctions (red) in the intercalated disc of murine cardiac muscle cells (Vanslembrouck et al., 2018). **(B3, B4)**: Tight junctions and desmosomes between SARS-CoV-2 virus infected cells **(B3)** and human lung epithelial cells **(B4)** imaged by FIB-SEM. **(B3)**: Two neighboring cells with a tight junction (yellow) at the contact site **(B3)** and a scanning EM (SEM) image of the tight junction seen as a dense structure between cells **(B3’)**. 3D reconstruction of neighboring cells seen in **(B3)**, with the tight junctions (yellow), small focal adhesions, likely desmosomes (purple) and virus particles (red) **(B3’’)**. **(B4)**: Desmosome-like junction between epithelial cells imaged by SEM is visible as a dense short structure and a 3D reconstruction of neighboring cells seen in **(B4)** with virus particles (red) and desmosome-like junctions (blue) **(B4’)** (Baena et al., 2021). Reproduced/adapted with permission.

This review aims to be a guideline for researchers in the field of intercellular junctions, to provide them with an overview of different imaging techniques and to help them select the best technique for the problem at hand. We outline the most relevant imaging techniques in increasing resolution order, including the advantages, limitations, and possibilities. Each technique includes examples of research that are used in literature to image tight junctions, adherens junctions and/or desmosomes (Figures 3, 4). This review does not focus on the technical in-depth explanation of each imaging modality. It is impossible to include all literature on this topic, and we picked significant studies and applications for this review. We apologize if we have missed some important research.

## 2 Light-Based Microscopy Techniques

### 2.1 Structural Information

#### 2.1.1 Light Microscopy

Bright-field illumination is one of the most widely used observation modes in optical microscopy. The illumination light is transmitted through the sample and contrast is generated by the absorption of light in dense areas of the specimen. This enables researchers to see the (sub)structures in the sample, for example, desmosomes in mucosa tissue (Raju et al., 2014). Due to the limited resolution of light (in theory, limited to 0.2 µm), light microscopy is often accompanied by electron microscopy (EM) images to confirm the findings. White light microscopy makes it efficient to get a fast and general overview of the intercellular junctions. Even though the resolution is lower, the field of view is larger, and the surrounding structures are visible. It is an easily accessible, low technical demanding technique that is widely used and available in every research laboratory.

Not only the ultrastructure of intercellular adhesions is of interest, but research has also been conducted to unravel the complex composition of the adhesion complexes, including the different proteins. This can be done by using fluorescence microscopy or EM combined with the labeling of the individual junctional proteins. For instance, protein labeling when using light and electron microscopic techniques was a critical part of the discovery of the area composita (a mixed type of junction at the intercalated disc of cardiac muscle cells) (Borrmann et al., 2006; Franke et al., 2006).

Imaging of cell junctions with light has challenges due to the limited spatial resolution of fluorescence microscopes (250 nm in xy and 600 nm in z). Another challenge is the size of the junctions, which is close to or below the resolution limit. Therefore, it is difficult to distinguish between different proteins within a junction, and proteins can appear co-localized when in reality their organization is distinct (Rayleigh 1879). Recent advances made it possible to push the lateral (xy) resolution of fluorescent imaging beyond 200 nm, up to the two digits nanometer range (Table 1).

**TABLE 1:** An overview of the imaging techniques used to study the structure and composition of cell-cell adhesions with light-based microscopy. SIM: structured illumination microscopy; SMLM: single-molecule localization microscopy; PALM: photoactivated localization microscopy; STORM: stochastic optical reconstruction microscopy; STED: stimulated emission depleted; PLA: proximity ligation assay; FRET: fluorescence resonance energy transfer.

Imaging technique	—	Resolution	Imaging depth	To investigate	Cons	*In vivo* imaging
Wide-field	—	xy: max 0.2 µm	2–5 µm	Fast and general overview, large field of view	Lower resolution	Possible
				Largely available		
				Technically not demanding		
Confocal	—	xy: 500–100 nm	1–10 µm	Localization of proteins in and around the intercellular junction complexes, how specific proteins behave in relationship to each other and the cytoskeleton	Lower resolution	Possible
		z: 500 nm		Easily accessible and widely used		
Super resolution	SIM	xy: 100–130 nm	Up to 20 µm	Sub-junctional protein organization of adherens junction and connection to cytoskeleton, co-localization experiments, actomyosin around junctions, link microtubuli and tight junctions	Needs some sample preparation optimization; Some technical handling	Possible
		z: 100–350 nm		Can image deeper in cell; easy set-up; conventional fluorescence dyes; 3D possible		
	STED	xy: 20–50 nm	Up to 20 µm	Co-localization, connection with actin cytoskeleton	Phototoxicity	Possible
		z: 100–300 nm		High resolution and deep inside the cell	Limited availability in optimal fluorophores	
		—		—	Needs sample preparation optimization and some technical handling	
	Expansion microscopy	xy: 70 nm; z: 70 nm	Based on imaging technique (confocal or super-resolution)	High resolution with accessible technique	Not yet widely used so effect of spatial changes of junctions during expansion unknown	Possible
	SMLM (including PALM and STORM)	xy: 20–50 nm	200 nm	Single molecules/proteins visible, sub-junctional protein localization, cytoskeleton-junction interface, identification and quantification of the protein organization of junctions, distances between proteins	Limited penetration depth	Possible
		z: 40–100 nm		Very high resolution	Needs sample preparation optimization	
		—		—	Some technical handling	
Co-localization techniques	PLA	Based on imaging technique used	Based on imaging technique used	Detect and quantify close protein interactions of proteins within 40 nm of each other at the intercellular junction	Based on imaging technique used	Based on imaging technique used
				Analysis with confocal or super resolution microscope		
	FRET	Based on imaging technique used	Based on imaging technique used	Protein interactions or co-localization of proteins within 8–10 nm from each other	Based on imaging technique used	Based on imaging technique used
					Construction sensors may be challenging	

### 2.2 Compositional Information

#### 2.2.1 Confocal Imaging (including Spinning-Disc and Point-Scanning)

**FIGURE 5:**
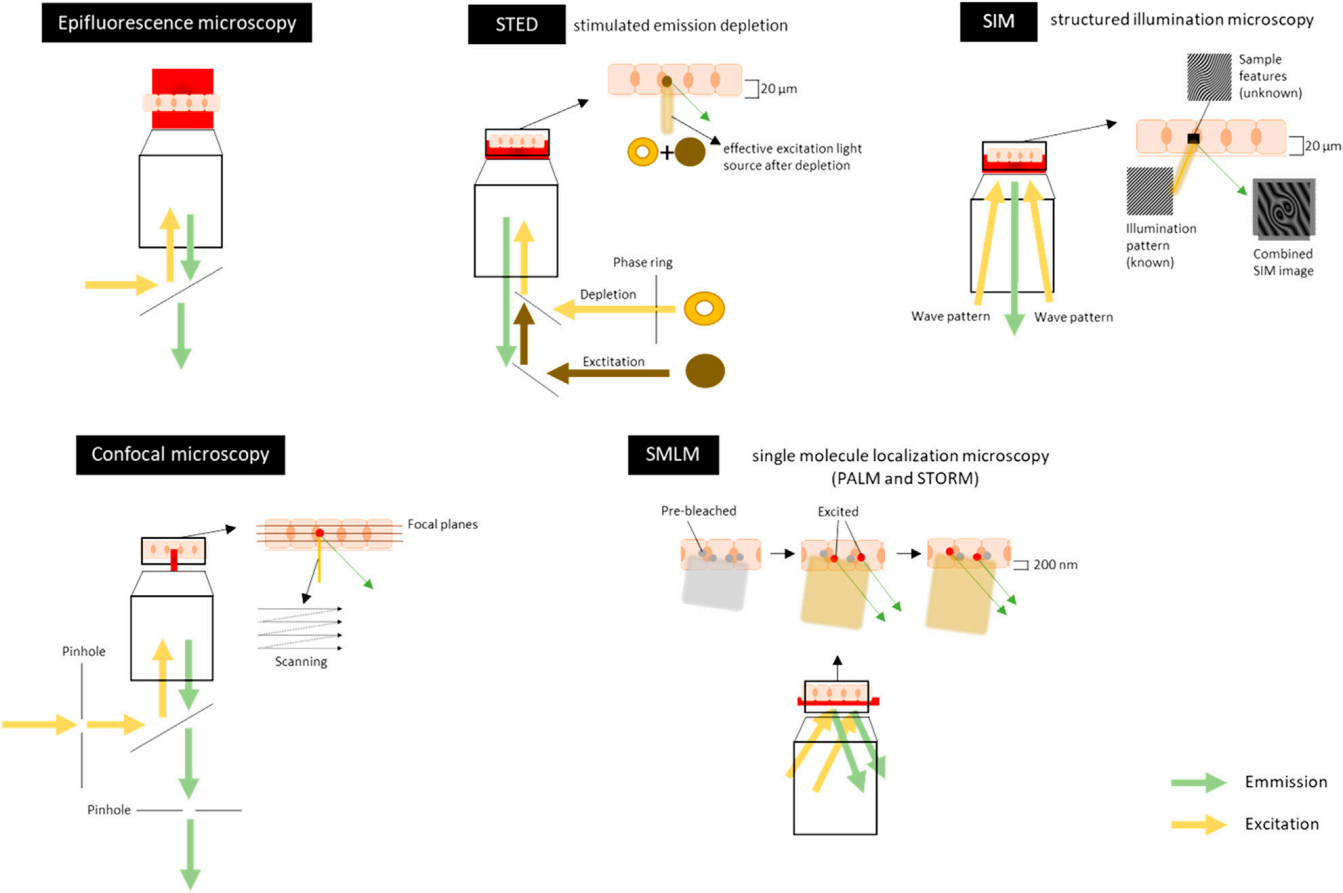
Schematic overview of visible light-based techniques for imaging the tight junctions, adherens junctions and desmosomes. Sample is represented here as a layer of cells, which can either be fixed (chemically or by cryo-freezing) or non-fixed (live-cell imaging). The sample can also be a tissue section. In Epifluorescence microscopy the illumination light is transmitted through the sample which excites fluorescent molecules in the stained sample. It visualizes the (sub)-cellular structures in a large field of view in the sample to get a general overview of the intercellular junctions. Confocal microscopy uses lasers to illuminate and scan the tissue at a certain depth (focal plane). Pinholes are present to physically block out-of-focus light and to eliminate or reduce background information away from the focal plane. Emitted fluorescence is recorded. Scanning can be done at each focal plane to make optical sections throughout the tissue (5–30 µm). The lateral (xy) resolution is 500–100 nm and the axial (z) resolution is around 500 nm. For an example of an image, see Figure 3A. Stimulated emission depletion (STED) microscopy uses two overlapping, synchronized lasers that raster scan over the stained sample; one to excite the sample, the other to deplete some of the excited fluorophores to the ground state. The depletion of the fluorescence is done in a donut shape. This allows the excitation of only a small volume of the labeled fluorescent proteins in the sample. Emitted fluorescence is recorded. Imaging can be done throughout the tissue (up to 20 µm thick). The lateral (xy) resolution is 20–50 nm and the axial (z) resolution is 100–300 nm. Structured illumination microscopy (SIM) uses high frequency stripe-patterned excitation (the illumination/wave pattern) to illuminate the sample containing a fluorescent dye attached to a structure of interest. Emitted fluorescence is recorded. The imaging can be done up to 20 µm into a sample and optical sections can also be made along the z-axis. The lateral (xy) resolution is 100–130 nm and the axial (z) resolution is 100–350 nm. For an example of an image, Figure 3C. Single molecule localization microscopy (SMLM) [includes PALM (photoactivated localization microscopy) and STORM (stochastic optical reconstruction microscopy)] sequentially excites random subsets of fluorophores labelled to the protein of interest. In general, the fluorophores have an ON/OFF mechanism allowing a sparse population of non-overlapping emitters. A wide variety of organic and fluorescent dyes and different colors can be used and combined to get multiplex of single molecules. Emitted fluorescence is recorded. The light penetration depth is limited and there is scattering light. Imaging can only be done in the first 200 nm of the sample. The lateral (xy) resolution is 20–50 nm and the axial (z) resolution is 40–100 nm. For an example of an image, Figure 3C. Figures are not on scale.

Confocal microscopy is a fluorescence imaging technique that uses lasers to illuminate and scan the tissue at a certain depth (called the focal plane) while pinholes are present to physically block out-of-focus light and thus eliminate or reduce background information away from the focal plane (Figure 3A and Figure 5). Emitted fluorescence is recorded, and optical sectioning of the specimen is obtained. Confocal microscopes may reach a resolution of 170–250 nm laterally (xy) and around 500 nm axially (z) (Ooshio et al., 2010; Elliott 2020; Valli et al., 2021) and relatively thick samples (5–30 µm) can be imaged. Thicker samples do produce more out-of-focus light because the objective lens does not have sufficient depth of focus resulting in the detection of light from sample planes above and below the focal plane. However, many new methods have emerged to overcome this and give better results in signal-to-noise ratio (SNR) and resolution, including pixel reassignment (Airyscan) (Murray 2011; Elliott 2020; Valli et al., 2021).

One example out of thousands of imaging intercellular junctions by confocal microscopy: research showed that claudins and junctional adhesion molecules (JAMs) are major cell adhesion molecules at tight junctions, whereas cadherins and nectins are major adhesion molecules at adherens junctions. Claudins and JAMs are associated with zona-occludens (ZO) proteins, whereas cadherins are associated with β- and α-catenins, and nectins are associated with afadin. To investigate how tight junction components are recruited to the apical side of adherens junctions during polarization of epithelial cells, researchers used confocal laser scanning microscopy to study the roles of afadin and ZO-1. Researchers saw that nectins first form cell-cell adhesions by recruiting the cadherin-catenin complex in order to form adherens junctions. This is then followed by the recruitment of the JAM-ZO and claudin-ZO complexes to the apical side of adherens junctions in order to form tight junctions (Ooshio et al., 2010). Before the formation of tight junctions, ZO-1 interacts with afadin. However, during and after formation of tight junctions, ZO-1 dissociates from afadin and is associated with JAM-A. Confocal microscopy was also useful to see that disruption of afadin impaired the formation of both adherens junctions and tight junctions while knockdown of ZO-1 only impaired the formation of tight junctions and not of adherens junctions (Ooshio et al., 2010).

Spinning disk technology, a fast confocal microscopy technique, employs a parallel array of pinholes on a rotating disk. With a similar resolution as in confocal microscopy (Zubkovs et al., 2018), this technology showed that epithelial apical junctions (tight and adherens junctions) of contractile tissue display a periodic assembly of bipolar non-muscle myosin II filaments that interlace with the peri-junctional actin and α-actinin (Figure 3A1) (Ebrahim et al., 2013). In that way, the structure forms a continuous belt of muscle-like sarcomeric units around each epithelial cell. The spinning disk illustrates that the sarcomeres of adjacent cells appeared to be precisely paired across the junctional line to create a transcellular contractile network as seen with transmission electron microscopy (TEM; see below) (Ebrahim et al., 2013).

Additionally, confocal microscopy elegantly showed that desmosomes stay intact and are crucial for the apoptotic cell extrusion in a monolayer of epithelial cells (Figure 3A2) (Thomas et al., 2020). Confocal imaging showed that upon apoptosis the formation of actomyosin cables occurred in the vicinity of desmosomal junctions and that they subsequently deviated from desmosomal junctions during its constriction, which coincided with a loss of straightness of the desmosomal junction.

Confocal microscopy is thus an ideal technique to study the localization of proteins in and around the intercellular junction complexes to see how specific proteins function and how they interact with each other. Confocal imaging is a very integrated technique, not technically demanding and readily available in almost every research lab. It is often used as a first approach to understand or unravel complex intercellular junctions.

#### 2.2.2 Super-Resolution Techniques

The progresses made to overcome the optical diffraction limit in the field of optical microscopy over the last few decades has greatly advanced the resolving power. This has opened windows for researchers to observe objects with much higher spatial resolution. Compared with wide-field fluorescence microscopy, super-resolution techniques offer a superb resolution to study the protein organization, protein dynamics and protein co-localization at nanoscale in macromolecular complexes. The resolving power of 20–120 nm (Table 1) (Hell and Wichmann, 1994; Neil et al., 1997; Betzig et al., 2006; Rust et al., 2006; Valli et al., 2021) also enables to distinguish between homogenous mixing and sub-junctional clustering, to see the relative position of proteins within the junction (proximal or distal to the membrane) and allows a precise identification and measurement of the size, distribution and composition of junctional clusters. The type of super-resolution microscopy depends on the researcher’s needs, which includes the spatial resolution, temporal resolution and ease of sample preparation. Thicker samples are still challenging to image due to light scattering and optical aberrations. Cautious data interpretation is also warranted when analyzing data at a nanometer resolution level. For example, researchers need to be aware that antibodies around 10 nm in size leave a 20 nm cloud of uncertainty around the object of interest (when labeling with primary and secondary antibodies). Other labeling techniques, including direct labelled primary antibodies, Fab fragments, nanobodies, affimers and chromobodies, might be considered as they reduce the distance between the protein and fluorophore (Bates et al., 2007; Carrington et al., 2019).

A variety of super-resolution microscopic techniques exist. Below we give a short description of the techniques, with their pros and cons, and some elegant examples of imaged adhesions (Figure 3C and Figure 5). Super-resolution techniques can be broadly split into two categories: super-resolved ensemble microscopy techniques, which improve the resolution of overall structures, and super-resolved single-molecule localization microscopy techniques (SMLM), which use localizations of individual fluorescent molecules to build up an overall structure (Valli et al., 2021).

##### 2.2.2.1 Ensemble Super-Resolution Techniques

1) Structured Illumination Microscopy

Structured illumination microscopy (SIM) is a technique that uses high-frequency stripe-patterned excitation (that is usually the frequencies beyond the resolving power of optical transfer function), to illuminate the sample containing fluorescent structures. The collected signals are mixtures of known and unknown information. The maximum frequency the system can resolve is limited by the diffraction system (Figure 5). In the case of SIM, the resolution can be enhanced by a factor of two (Zhao et al., 2021). The excitation lines are spatially restricted so multiple images can be collected from the excitation pattern with different phases and orientations after which they can be reconstructed. The technique has a lateral resolution (xy) of 100–130 nm (up to 50 nm) and an axial (z) resolution ranging between 100 and 350 nm. The imaging can be done up to 20 µm into a sample, but the resolution is decreasing with increasing depth of imaging (Heintzmann and Huser, 2017; Bartle et al., 2018; Wu and Shroff, 2018; Gonschior et al., 2020; Bond et al., 2022). Recent developments applying adaptive optics (Ji 2017) with SIM enable imaging with 150 nm lateral and 570 nm axial resolution at a depth of 80 µm through the *C. elegans* (Lin et al., 2021). One of the advantages of SIM is that it is a relatively simple and straightforward optical setup. This technique enables researchers to image biological samples with conventional fluorescence dyes that make it readily accessible. Without manipulating the fluorophores’ physical chemistry properties, multiple fluorescent tagging of the target of interest is practical and therefore, *in vivo* imaging to monitor protein co-localization is one of the benefits of using SIM. Another benefit would be the low photo-toxicity compared with other super-resolution techniques (Bartle et al., 2018; Gonschior et al., 2020). Furthermore, 3D-SIM can be expanded from 2D-SIM by using three beams of interfering light. The high-frequency pattern generated along the z-axis not only improves the axial (z) spatial resolution, but also makes z-sectioning possible. Two-photon/non-linear microscopy has made the optical sectioning even better by excluding the light scattering (Wu and Shroff, 2018; Manton et al., 2020). However, this can lead to more phototoxicity and slower temporal resolution. A way to decrease the phototoxicity is to use a cryo-temperature set-up which also increases the fluorescence lifetime drastically (Le Gros et al., 2009). Speeding up the data acquisition can also be acquired by the use of spatial light modulators (Han et al., 2019). Disadvantages of SIM are the lower enhancement of resolving power compared to the other techniques described below, more noise when imaging thicker samples, artifacts when there is a refractive index mismatch, reconstruction artifacts and 3D-SIM can suffer from limited background rejection.

SIM has, for instance, been used to unravel the sub-junctional protein organization of adherens junctions and their connection to the cytoskeleton in endothelial cells. Researchers showed that both E-cadherin and nectin are localized in separate clusters within one adherens junction but their cluster size and distribution differ significantly and are independent of each other (Figure 3C3) (Indra et al., 2020).

Spinning-disk confocal microscopy showed that the non-myosin II protein makes up the connection with the actin cytoskeleton next to the epithelial junction, forming the actin—non-myosin II peri-junctional network (see part 2.2.1). SIM experiments confirmed this and a continuous cortical actin ring with underlying sarcomeric-like non-muscle myosin bipolar filaments in the region next to apical cell-cell junctions could be seen (Ebrahim et al., 2013; Van Itallie et al., 2014; Choi et al., 2016). More researchers using SIM showed that two non-muscle myosin II isoforms differentially regulate the biogenesis of the adherens junctions through their association with distinct actin networks and show association with distinct pools of the actin network (Heuzé et al., 2019).

From confocal microscopy imaging, ZO-1 is an important protein of the epithelial adherens junctions to maintain tissue homogeneity (see part 2.2.1). SIM experiments showed that ZO-1 also has a crucial role in the contractile activity of the actomyosin complex around the junctions. At tricellular junctions, where each bicellular border is an independent contractile unit, SIM showed that the borders are anchored end-on to cadherin complexes with actin cables (Choi et al., 2016). When combining SIM, proximity assays (see part 2.2.3.1) and force experiments, researchers could reveal that ZO-1 exists in stretched and folded conformations within epithelial cells, depending on the actomyosin-generated force (Figure 3C6) (Spadaro et al., 2017).

SIM can also be used to show the link between microtubules and tight junctions. Researchers discovered a planar apical network of microtubules just beneath the apical plasma membrane, at the same level as the tight junctions. This network could not be clearly identified by conventional immunofluorescence microscopy. 3D cell cultures imaged by SIM helped the researchers to analyze the biological relevance of microtubule-tight junction association. For example, the cytosolic protein cingulin at tight junctions interacts with microtubules, which has a crucial role in maintaining the proper epithelial morphogenesis of the junctions (Yano et al., 2013).

The high resolution of SIM also allowed researchers to see the colocalization of desmoglein3 (Dsg3) with lipid raft markers in cultured epithelial cells (Stahley et al., 2014), which together with SIM of patients biopsy tissue and biochemistry experiments enabled to understand the desmosome dynamics and pathogenesis of the autoimmune disease Pemphigus Vulgaris (Stahley SN. et al., 2016) (Figure 3C2). They identified the desmosomes “split” along with the adhesive interface at blister sites and observed that mechanical stress on this can lead to desmosome splitting.

2) Stimulated Emission Depletion

Stimulated emission depletion (STED) microscopy uses two overlapping, synchronized lasers that raster scan over the sample; one to excite the sample, the other to deplete some of the excited fluorophores to the ground state. The depletion of the fluorescence is done in a donut shape. This allows the excitation of only a small volume of the labeled fluorescent proteins in the sample. The depletion laser can work continuously or in pulses with the excitation laser, both having advantages and disadvantages (reviewed in (Valli et al., 2021)). The lateral resolution (xy) is 20–50 nm while the axial (z) resolution is 100–300 nm (70 nm for 3D STED) (Hell and Wichmann, 1994; Rittweger et al., 2009; Valli et al., 2021; Bond et al., 2022) over the whole sample of 20 µm thick (Vicidomini et al., 2018). Confocal-like STED techniques automatically excludes out-of-focus signals, however, the aberration issues deteriorate as penetration gets deeper. For imaging thicker samples, this issue can be tackled by applying adaptive optical devices (Gould et al., 2012). Both multicolor and live imaging is possible, enabling the imaging of tight and adherens junction components and the actin cytoskeleton with <60 nm resolution deep inside cells (Maraspini et al., 2019). Phototoxicity could be one of the issues that STED microscopy will suffer from, if long-term live-cell imaging is needed (Bottanelli et al., 2016). Therefore, the experimental approach should be carefully considered (Kilian et al., 2018). Another drawback of STED is the limited availability of bright and photostable dyes with an optimal emission intensity at the depletion laser wavelength so optimal label density is needed for accurate interpretation of the labeling data [reviewed in (Gonschior et al., 2020)]. STED can be used in combination with atomic force microscopy (Curry et al., 2017) and photobleaching techniques for an additional dimension in unraveling the molecular organization and dynamics of complexes (Gonschior et al., 2020). The ultrastructural preservation of living cells and tissues can be compared in several conditions (Schnell et al., 2012) and should be taken into consideration when analyzing and comparing data.

STED has been used to study cell-cell adhesion complexes in the apical and lateral membrane domain of the epithelial cell layer and organoids (cysts), respectively (Maraspini et al., 2019). In this study, researchers used a technique called inverted filter mounting. This inverts the 2D epithelial monolayer and enables the access of the apical membrane with STED imaging. With 3D cell culture imaged by STED, researchers resolved cell-cell adhesion complexes in the lateral membrane; i. e., single E-cadherin clusters in relation to filamentous actin (F-actin) were visible in the lateral membrane showing that E-cadherin clusters are larger than 200 nm and often elongated. F-actin did not precisely co-localize with E-cadherin but formed more of a filament surrounding E-cadherin. That is similar to the one seen in 3D stochastic optical reconstruction microscopy (STORM) data of ectopically expressed E-cadherin in epithelial monolayers (as described below (Bertocchi et al., 2017)).

Fuchs et al. demonstrated that Pkp regulates the clustering of desmosomal cadherins in keratinocytes as an isoform-specific manner (Fuchs et al., 2019). Both Pkp1 and Pkp3 are required for junctional membrane availability of desmosomal cadherins Dsg1 and Dsg3. In contrast, Dsg3-snap clustering, as shown by STED imaging, is a specific function of Pkp1.

3) Expansion Microscopy

Expansion microscopy is a newly developed imaging technique that achieves nanoscale precision for imaging specimens at ∼70 nm lateral (xy) resolution. For this, chemically processed biological samples are embedded in a matrix of swellable polymers, digested and expanded isotropically (∼4.5× linear expansion). Immunolabeling can be done before or after the expansion. Tissue is then imaged by a fluorescent light microscope, enabling imaging at the super-resolution range using conventional diffraction-limited microscopes (Chen et al., 2015; Rowland et al., 2015; Margineanu et al., 2016; Culley et al., 2018). Take the *C. elegans* for example, researchers visualized adherens junctions proteins seen as a pattern of longitudinal lines spanning across the entire animal at nanometer resolution [antibody staining against DLG-1 (disc large; adherens junction protein)] using expansion method using a confocal microscope (Yu et al., 2020). This could be a readily available and easily accessible technique for many researchers as there is no need for specialized equipment/setup. However, one has to cautious using expansion microscopy for imaging and subsequent analysis of intercellular junctions. The expansion of the tissue may change the spatial relationship between proteins leaving the researcher unsure if it is a reflection of their original position *in vivo* (Gallagher and Zhao, 2021).

##### 2.2.2.2 Single-Molecule Localization Microscopy

SMLM is a super-resolution technique that, not exclusively, includes photoactivated localization microscopy (PALM) and STORM (Sauer and Heilemann, 2017; Lelek et al., 2021). SMLM methods apply sequentially excitations among random subsets of fluorophores followed by computing their positions. SMLM uses the fluorophores ON/OFF mechanism allowing a sparse population of non-overlapping emitters; i.e., fluorophores that are too close together (in subdiffraction distances) and cannot be differentiated when fluorescing at the same time, can be excited separately one by one (see Figure 5). There are many variations of SMLM based on the types of fluorophores used and how the activation/deactivation is used. For example, STORM uses specialized buffers to drive standard organic fluorescent molecules into long-lived dark states, in which fluorophores cannot be excited before returning to the ground state (Sauer and Heilemann, 2017; Lelek et al., 2021). Under these circumstances, optimizing buffer composition is thus crucial. PALM, on the other hand, uses specific photoswitchable/blinking fluorophores (often genetically encoded) to achieve stochastic activation so that only a subset of fluorophores is in ON state at given time.

The most attractive factor of using SMLM to study biological questions would be the high spatial resolution. The achieved resolution is 20–50 nm lateral (xy) and 40–100 nm axial (z) (Betzig et al., 2006; Rust et al., 2006; Bond et al., 2022). Moreover, SMLM allows the use of a wide variety of organic and fluorescent dyes and different colors which can be combined to get multiplex, live cell imaging of single molecules. It also allows direct genetic labeling with photoactivatable proteins or self-labeling enzymatic tags. Endogenous labeling of junctional components can also be performed, preferably with polyclonal antibodies that bind multiple epitopes (Gonschior et al., 2020). The high resolution in which researchers can localize individual molecules allows the observation of individual claudin strands or E-cadherin nanoclusters. When SMLM is used in total internal reflection fluorescence (TIRF) mode, analysis of individual adherens and tight junction proteins formed in the lower plasma membrane is possible. The spatial resolution is drastically dominated by the SNR. This is a major drawback when imaging thicker samples (more than 200 nm) due to the light penetration depth and the scattering light (Gonschior et al., 2020). Therefore, it is important to mention that SMLM imaging is currently limited to shallow sample depths. Moreover, the stability of fluorophores is extremely variable and depends upon experimental conditions (Endesfelder et al., 2014). Multicolor imaging of SMLM is tricky due to point-spread-function overlapping as a result of high-density labeling. High activation probability is needed for higher temporal resolution for imaging live cells. Proper data analysis of SMLM data can also be challenging (Bond et al., 2022). More info about SMLM techniques can be found in several good reviews (Lelek et al., 2021; Valli et al., 2021).

STORM revealed that tight junctions in primary alveolar epithelial cells are discrete punctate structures (called tight junctions spikes) rather than a continuous network observed by conventional fluorescence microscopy. In addition, they showed sub-junctional proteins remodel in response to biochemical environmental changes (Schlingmann et al., 2016). Specifically, the authors saw a decreased claudin-18 co-localization with ZO-1 but increased claudin-18 and claudin-5 co-localization, causing a reduced barrier function and impaired tight junction function. Sample preparation has to be taken into consideration, however, as thin, alveolar epithelial cells were cultured and mounted on glass coverslips. These cells are squamous and have a limited tight junction mesh network compared to other epithelial cells, therefore results can differ. The super-resolution of the technique enabled the visualization of these changes which are often <500 nm^2^ small. With the lateral (xy) 50 nm resolution in STORM, individual claudin strands can be visualized; confirmation differs from the claudin strands detected by freeze-fracture electron microscopy (FFEM) (Kaufmann et al., 2012), but it can be challenging to image native claudins in cuboidal epithelia as it also requires super-resolution in the z-axis. More changes in the morphology of tight junctions observed by super-resolution do not necessarily correlate with changes in ultrastructure (Lynn et al., 2020).

Previous conventional microscopy showed distinct E-cadherin clusters in the apical adherens junctions. However, 3D SMLM looked into more detail to this E-cadherin clustering using both cell cultures and *in vivo* models to study mature adherens junctions at a high resolution (Figure 3C5) (Truong Quang et al., 2013; Wu et al., 2015). The investigators observed that the size and shape of these E-cadherin clusters are similar as in the lateral junctions, but less closely spaced compared to the apical clusters. Next to this different surface distribution, researchers also found that the protein density is much higher in adhesive clusters compared to non-adhesive ones. In addition, both the cytosolic and extracellular part of E-cadherin plays an important, but not exclusive, role in the clustering of E-cadherin (Wu et al., 2015). The researchers imaged apical and lateral E-cadherin-based adhesions at a depth of 0.3–1 µm. Therefore, the results of this study have to be interpreted with caution as lateral junctions are often found deeper in the tissue, depending on the cell type. This can lead to speculations that many lateral junctions could not be imaged because of the limited illumination depth of STORM/PALM technique. The cytoskeleton-junction interface can also be studied with SMLM techniques. The nanometer resolution of SMLM makes it possible to study the E-cadherin clusters in junctions and their interaction with actin filaments during biological processes, such as endocytosis. Truong et al. fused a photoconvertible monomeric fluorescence protein to E-cadherin and knocked it into the cell line to replace the endogenous E-cadherin. They noticed that this E-cadherin localized into apical adherens junctions during the gastrulation stage, with a 30 nm precision in the plane of the epithelium and 50–100 nm precision along the apicobasal directions (optical axis) (Truong Quang et al., 2013). 3D STORM was also able to show an F-actin meshwork surrounding lateral E-cadherin clusters as well as that the E-cadherin clusters and F-actin are positioned in the same z-plane. The researchers claim these observations are visible because the relative positions of E-cadherin and F-actin of the apical junction are shifted compared to the lateral clusters. They observed that the observation angle relative to the membrane is shifted by almost 90° (Wu et al., 2015). In a single isolated ventricular myocyte, Cerrone et al. showed by direct STORM (dSTORM) the relationship between adherens junctions protein N-cadherin and the microtubule plus end, and its nanoscale retraction in situations with mutated desmosomes (see Fig. 8 in Cerrone et al., 2014).

Interferometric PALM (iPALM) combines photoactivated localization microscopy with single-photon, simultaneous multiphase interferometry to provides sub-20-nm 3D protein localization with optimal molecular specificity (Shtengel et al., 2009). iPALM together with 3D STORM showed the focal adhesions in epithelial cells with <20 nm axial (z) resolution. This revealed a multi-compartment architecture with the plasma membrane-proximal compartment segregated from the actin cytoskeleton, while bridged by an interface zone containing vinculin (Kanchanawong et al., 2010). Because of their natural localization between the cell membrane and the extracellular matrix often facing the coverslip, focal adhesions are ideal objects to image with SMLM techniques. Bertocchi et al. imaged adherens junction proteins by iPALM, but because of the limited imaging depth, they imaged epithelial cells cultured on a planar E-cadherin coated substrate format in which the cells form cadherin-based adhesions (Bertocchi et al., 2017). It has to be noted that the junctions are not in their native state, however this technique allowed the researchers to clearly resolve the dorsal and ventral plasma membranes, with the z-position of the latter at 30–40 nm above the substrate. F-actin bundles were found at a higher z-position, centering around 70–80 nm with an angle of approach nearly parallel to the adhesion plane. They illustrated that an activation-induced conformational change in vinculin of around 30 nm resulted in bridging both compartments and impacted the localization of several actin-regulating proteins (such as zyxin and VASP) at the same time (Figure 3C4) (Bertocchi et al., 2017). Even though the junctions are not located in their native location and their physiological relevance could be a point of discussion, this engineered approach increased the understanding of the relationship between structure and mechanical integration in adherens junctions.

The high resolution of dSTORM is ideal to identify and quantify the protein organization of desmosomes. SIM and dSTROM elucidate the plaque mirror symmetry, desmosomal plaque length and plaque-to-plaque distance in epithelial cells (Figure 3C2) (Stahley S. N. et al., 2016). Results show that desmoplakin is further localized from the plasma membrane than then previously observed with immunogold studies; i.e., desmoplakin is oriented with its long axis at an angle in the plaque and not perpendicular to the plasma membrane. This changed the view of the protein arrangement within desmosomes. It indicates that the desmosome molecular architecture and organization of plaque proteins is critical for desmosome function and correlates the protein organization within the desmosomes with changes in adhesive strength. PKP is a protein known to be affected in multiple diseases and the PKP-1 isoform is known to promote desmosome formation by recruiting and clustering desmosomal proteins. Overexpression of this isoform resulted in the presence of hyper-adhesive desmosomes (Hatzfeld et al., 2000; Bornslaeger et al., 2001). In this PKP-1 induced hyper adhesive state (Tucker et al., 2014), an increased desmosomal length occurs and plaque proteins are reorganized, such as an orientation change of desmoplakin shown by dSTORM (Figure 3C2) (Stahley SN. et al., 2016).

Minimal photon flux imaging (MinFlux) is a recently developed method that combines SMLM techniques with STED; i.e., fluorophores blink or switch as in STORM and PALM but a donut-shaped beam excites the tissue used in STED. Therefore, fluorophores that are exactly in the middle of the beam will not be excited which can then be used to precisely locate the proteins of interest (Gwosch et al., 2020). MinFlux reaches a resolution of 1–3 nm for structures in fixed and living cells. This nanometer resolution technique has been used to study nanoclusters in synapses, which thus could be a potential method to look at the higher-level organization of nanoclusters in intercellular junctions (Wu et al., 2015). For instance, one could look at the higher-level organization of E-cadherin at the free membrane as well as at cell-cell junctions. The latter has been done with ectopic expressed E-cadherin in *Drosophila* (Chandran et al., 2021). Even though MinFlux is a powerful technique with a high localization precision and very high resolution, it is a computationally intensive technique and not yet widely available.

To summarize, the lateral (xy) resolution of super-resolution microscopy techniques can vary from 50 to 120 nm and can even go up to 20 nm (or 1 nm with the recently developed MinFlux), while the axial (z) resolution varies between 50 and 300 nm. As native intercellular junctions extend in the axial (z) direction in polarized monolayers and are found several micrometers away from the glass surface, optical sectioning of both STED and SIM and the possibility to increase the resolution in the z direction makes these techniques useful to study the junctional structures in a native context. The resolution combined with the imaging depth in the sample, which varies from 20 microns to 200 nm, mainly decides what technique you want to use for your research question. With an axial (z) resolution of sub 100 nm and a high penetration depth, SIM allows researchers to image deep into the sample with a fast speed possible in live cells. This commercially available imaging technique is not too expensive and often used to look at sub-junctional protein organization of adherens junctions and connection to the cytoskeleton. Moreover, researchers performed colocalization experiments, studied the actomyosin around junctions and unraveled the link between the microtubules and tight junctions using STED. The technique also allows imaging up to 20 microns deep in the sample but has a higher axial (z) resolution compared to SIM. However, some phototoxicity can occur in live cell imaging due to the high laser power and there is limited availability of fluorophores. STED is a highly commercially available technique that can be expensive. It has been used to study the co-localization of proteins and to understand the connection of the junctions with the actin cytoskeleton. In contrast to SIM and STED, SMLM cannot image deep into the cell, only thin samples can be used, but it can image at a 20–50 nm axial (z) resolution which allows the identification of single proteins. Therefore, SMLM is used to study sub-junctional protein localization and cytoskeleton-junction interface. If proteins are in the same plane, it can also be used to identify and quantify protein organization of junctions and measure distances between proteins in and around the intercellular junctions. Overall, super-resolution microscopy is an accessible imaging technique that gives highly detailed information on the localization and dynamics of individual intercellular proteins. Carefully optimizing sample preparation and imaging setup is crucial for correct imaging and interpretation of the data. Access to these super-resolution technologies can be challenging and the use of core facilities is often warranted here to get the correct expertise.

#### 2.2.3 Using Confocal and Super-Resolution Techniques to Determine the Co-Localization of Proteins

Intercellular junctions are complex multi-protein structures that are densely packed to provide inter- and intracellular communication as well as adhesion and structural integrity for proper homeostasis. To resolve these junctions in detail, often researchers want to study their exact interaction with one another. Super-resolution techniques provide the necessary lateral (xy) and axial (z) resolution to accomplish this, when combined with proper labeling and cautious interpretation of the data. Proximity assays and Forster resonance energy transfer (FRET) imaging are two techniques that have been used to unravel the protein network of tight junctions, adherens junctions and desmosomes.

##### 2.2.3.1 Proximity Assays

To see if two, or more proteins (RNA, DNA) are in close proximity, researchers use proximity-based assays. It can be used for the precise detection and quantification of proteins, protein interactions and modifications in different substrates, from fixed cells to tissue samples (Gullberg and Andersson, 2010). Proximity labeling-based methods coupled with mass spectrometry offer a high-throughput approach for systematic analysis of spatially restricted proteomes. It also helps to understand the cellular organization as well as interactome networks (Bosch et al., 2021).

For instance, to unravel the protein network of tight junctions, researchers fused biotin ligase (BirA) to the tight junction protein ZO-1 and looked which proteins are within its molecular dimension (Van Itallie et al., 2013). By identifying the resulting biotinylated proteins from mass spectrometry, this study provided a rich inventory of proteins and potential novel insights into functions and protein networks of tight junctions. This method was applied by the same research group that fused biotin ligase to occludin and claudin-4 in order to biotinylate their proximal proteins (Fredriksson et al., 2015) but also to E-cadherin (Van Itallie et al., 2014) in cultured epithelial cells. Other proximity proteomics experiments revealed a similar cellular environment at the nanoscale of adherens junction proteins. The latter can be spatially separated from the more basally located apical tight junctions (Baskaran et al., 2021). Another approach developed by Tan et al. makes use of APEX-mediated proximity labelling in polarized epithelial cells to generate a junctional proximity proteome (Tan et al., 2020). The researchers linked APEX2 peroxidase to a protein of interest, in this case tight junction-associated proteins, and the sample is then incubated in biotin phenol and H_2_0_2_ (instead of DAB and H_2_0_2_ as used for TEM (see section 3.3.1); biotinylates protein in a ∼20 nm radius) followed by fluorescently tagged streptavidin antibody for fluorescence microscopy. By using an optimized protocol, the associated proteins could be detected by mass spectrometry. Another assay is called proximity ligation assay (PLA) which is based on the system that secondary antibody probes have a short sequence-specific DNA strand attached to it. When two proteins are closer than 40 nm, the oligonucleotides that are bound to a protein-specific antibody form a circular template which is subsequently amplified and detected by complementary labeled oligonucleotide probes. Detection of proteins or protein complexes can be seen as countable distinct spots by either fluorescent microscopy (in case of the fluorescent label) or by brightfield microscopy (when horseradish peroxidase is used). This is helpful to study the interactions of cell surface proteins on two different cells (Sable et al., 2018). Researchers performed PLA with confocal and super-resolution imaging (SIM) to characterize the subcellular compartment where nectin-afadin and cadherin-catenin complexes in epithelial cells interact. The experiment confirmed that the interaction between nectin and cadherin complexes predominantly occur either at the periphery or on the outside of the cadherin-enriched clusters indicating a mosaic organization of the adherens junctions with an array of cadherin-catenin and nectin-afadin adhesive clusters (Figure 3B) (Indra et al., 2013).

To have a higher precision of co-localization, DNA labels (docking strands) can be anchored to the proteins of interest. Labeled protein pairs can be imaged with super-resolution microscopy. This technique, called point accumulation imaging in nanoscale topography (DNA-PAINT), has been used to image ryanodine receptors and alpha-actinin protein which is part of the cytoskeleton in cardiac tissue (Clowsley et al., 2020). DNA-PAINT could thus be expanded to investigate other cell adhesion molecules.

The proximity assay, with confocal or super-resolution imaging, is an adequate technique to precisely detect and quantify close protein interactions of proteins at the intercellular junctions. As SMLM techniques give the best lateral (xy) resolution, this is often the preferred imaging method. But this limits the imaging capabilities due to the limitation in illumination depth. It is an accessible technology that can be performed in any research lab with confocal or super-resolution microscopes available. A good balance between resolution and imaging depth, and thus the capability to image junctions in cell layer/tissue, needs to be considered.

##### 2.2.3.2 Forster Resonance Energy Transfer

Co-localization is often based on fluorescence imaging of multi-color fluorescent proteins to see if they are adjacent and can interact with each other. This is, however, still limited to the spatial resolution of the fluorescence produced by the fluorophores. FRET microscopy overcomes this limitation to determine the spatial proximity of single protein molecules in living cells as FRET only occurs when the distance between two approximately positioned fluorophores is 8–10 nm or less. Therefore, this is an ideal technique to study the interaction of molecules located within nanometers from each other. More technical information can be found in the literature (Shrestha et al., 2015). Finding a suitable method for labeling specific intracellular proteins with the appropriate fluorophores is difficult but recent developments, including biosensors (a single genetically-encoded construct) (Austen et al., 2013), can tackle this. Sample thickness and obtained resolution depend on the imaging tool used to visualize the FRET event, which is often a fluorescent microscope.

Recently, researchers unraveled an unexpected mechanism of cadherin oligomerization in cells by using FRET microscopy (Vu et al., 2021). It was thought that only extracellular domain interactions were responsible for lateral (*cis*) cadherin oligomerization (Singh et al., 2017; Thompson et al., 2020). However, FRET measurements showed that in adherens junctions, E-cadherin forms *cis* dimers at the plasma membrane and that the intracellular binding of p120catenin is crucial for this cadherin dimerization. Disrupted p120 catenin binding to E-cadherin further showed that this reduced cadherin *trans* binding affinity and cell adhesion. This implies that both extra- and intracellular cadherin domains play a role in the cadherin clustering and adhesion with p120 catenin as a key role (Vu et al., 2021). Another research study also used FRET to look at the *cis* and *trans* interactions, and their cooperativity, of cadherin transmembrane proteins. They found that the presence of *cis* interactions improved the lifetime of *trans* interactions between epithelial-cadherin extracellular domains, and vice versa, primarily due to allostery (Thompson et al., 2021).

FRET is useful to detect interactions of protein or co-localizations within 8 or 10 nm from each other. Recent developments are made to enhance or complement FRET with super-resolution techniques to increase the sensitivity of studying molecular proximities (Stöhr et al., 2012; Lee et al., 2017; Deußner-Helfmann et al., 2018), for instance a combination of FRET with STED (Szalai et al., 2021).

## 3 Electron and X-Ray Based Microscopy Techniques

To understand the cellular basis of human health, researchers simply look at the morphology of the sample in the healthy and diseased state. The membrane structure on which the intercellular adhesion complexes reside can be visualized without staining for specific proteins; and, based on the intermembrane distance and density of the junctional complexes, one can distinguish the three major junctional complexes. Several imaging studies are discussed to look at the structure of the cell-cell adhesions in two and three dimensions, also Table 2.

**TABLE 2:** An overview of the imaging techniques used to study the structure and composition of cell-cell adhesions with electron and X-ray microscopy; TEM: transmission electron microscopy; FFEM: freeze-fracture electron microscopy; PREM: platinum replica electron microscopy; ET: electron tomography; VEM: volume electron microscopy; SBF-SEM: serial block face scanning electron microscopy; FIB-SEM: focused ion beam scanning electron microscopy; SXT: soft X-ray tomography; immuno-EM: immuno electron microscopy.

Imaging technique	Resolution	Sample thickness	To investigate cell-cell adhesion and availability	Cons	*In vivo* imaging
TEM	xy: 2 nm	60–80 nm	Ultrastructure of the tissue at a nanometer resolution, morphology of junctions, relationship with the actin cytoskeleton, measuring intermembrane space	Some technical handling and sample preparation optimization	Not possible
			Correlation with LM possible		
			Easily accessible and widely used		
			FFEM and PREM possible		
ET	xy: 2 nm	Up to 300 nm	Filament arrangement within junctions, binding interactions between substructures, quantitative measurements	Some technical handling and sample preparation optimization	Not possible
			Easily accessible and widely used		
VEM (including SBF-SEM and FIB-SEM)	xy: up to 5 nm	Up to 20 µm per day	Junctions and ultrastructure in volume, quantification possible, relationship between different junctions and its localization on the membrane surface	Technically demanding and sample preparation optimization necessary	Not possible
	z: up to 5 nm			Limited availability, expensive machines	
SXT	xy: 10–50 nm	10 µm	Junctions at a nanometer resolution in the near-native state and possible to reconstruct the ultrastructure of the cell in 3D; Correlation with LM possible	Technically demanding and sample preparation optimization necessary	Not possible
	z: 10–50 nm			Limited availability because need for synchrotron	
Immuno-EM	Same as TEM	Same as TEM	Individual proteins and the ultrastructure of the sample in a nanometer resolution	Some technical handling and sample preparation optimization	Not possible
			Easily accessible and widely used		

### 3.1 2D Structural Information

#### 3.1.1 Transmission Electron Microscopy

**FIGURE 6:**
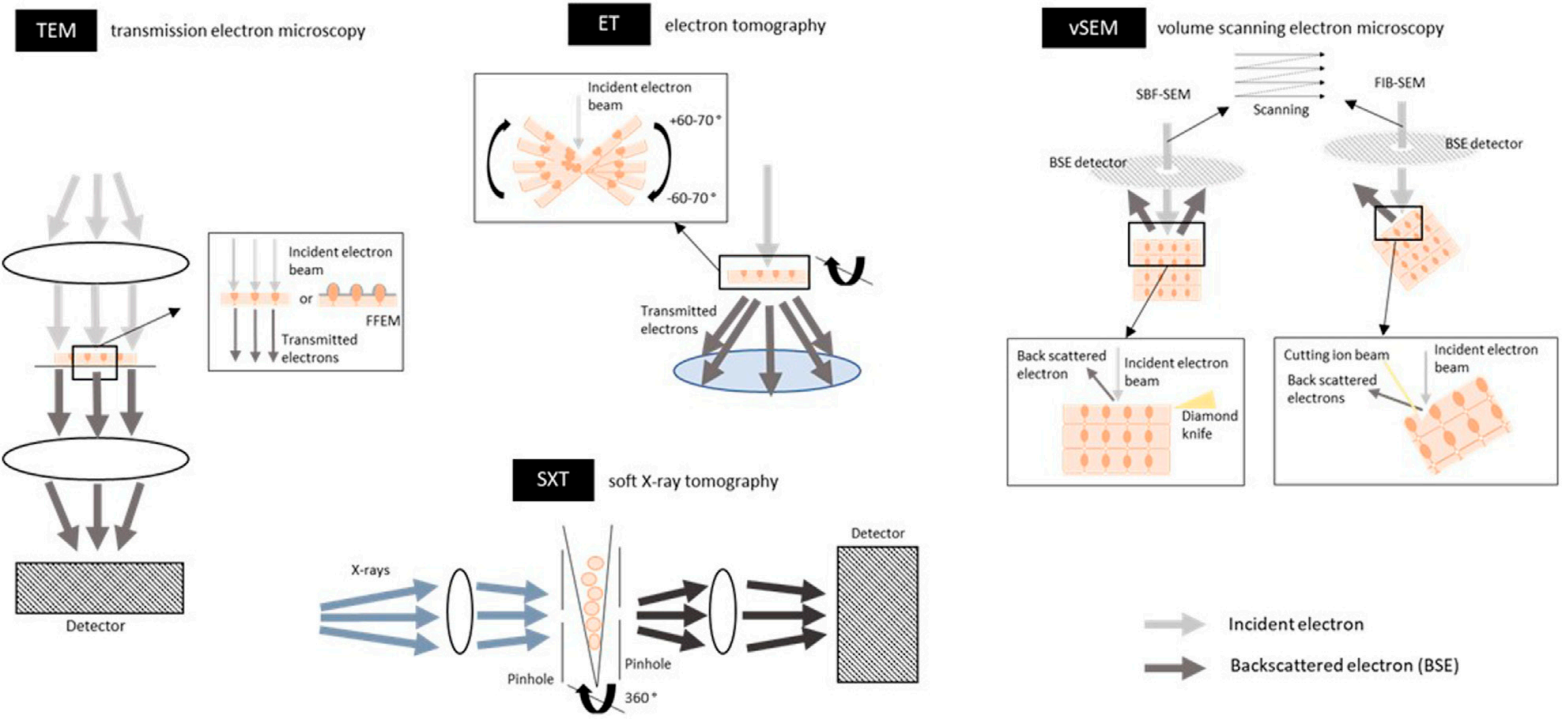
Schematic overview of electron and X-ray based techniques for imaging the tight junctions, adherens junctions and desmosomes. Transmission electron microscopy (TEM) uses an electron beam that passes through the sample (thin sections of 60–80 nm of tissue or cultured cells; either fixed or cryo-frozen because of vacuum conditions). The electron beam is absorbed and scattered by the sample which in their turn are captured on a detector and a contrast image of the sample can be seen. Electrons can only penetrate a very thin sample. In freeze fracture EM (FFEM) frozen biological samples are physically broken apart and platinum-carbon is used to replicate and contrast the fracture plane, which is then analyzed by TEM, exposing the intercellular junctions between cells. The lateral (xy) resolution is 2 nm. For an example of an image, see Figure 4A. Electron tomography (ET) is an EM-derived technique and is based on a tilt series of 2D images acquired at different viewing angles, which are subsequently aligned and combined digitally into a 3D volume. Tilt series contain projections from a complete ±60–70° rotation of the object to include projections from all possible directions. This gives a 3D view through the depth of the specimen at a high resolution and the intercellular junctions can be reconstructed in 3D. The sample must be thin sections (tissue or cultured cells; limited to about 300 nm; fixed or cryo-frozen) to render them electron transparent. The lateral (xy) resolution is 2 nm. For an example of an image, Figure 4B. Volume scanning electron microscopy (volume-SEM) can image larger volumes with a greater sample depth compared to ET. The technique is based on the alternation between scanning the tissue surface with a focused electron beam followed by cutting off a section of the *en-bloc* tissue. The electron interact with the tissue surface and the backscattered electrons (BSE) are then detected by a detector. Cutting the section is done by either a diamond knife [serial block face (SBF)-SEM] or an ion beam [focused ion beam (FIB)-SEM], enabling the cutting of sections with a thickness of 25–50 and 5 nm respectively. The maximum sample width for SBF-SEM is around 1 mm and 20–100 µm width for FIB-SEM. The sample preparation often includes heavy metal staining for contrast, embedding in plastic resin and coating of the sample with a thin layer of conductive material (i.e. gold) to reduce the charging effects. The resulting 2D stack of images can be then 3D reconstructed to see the intercellular junction in a volume. The voxel resolution is up to 5 nm. For an example of an image, see Figure 4B. Soft x-ray tomography (SXT) uses soft X-rays as an illumination light source. Projections are taken from different angles around a sample (cells in suspension or on a grid; cryo-frozen) by rotating the sample along its long axis. SXT image the sample in the water window, meaning that X-rays are absorbed by carbon and nitrogen in biological tissue more than by oxygen in water, resulting in grayscale that can be used for quantitative analysis. Soft X-rays have a limited penetration into the sample thus only samples of 10–20 µm can be imaged. The voxel resolution is 10–50 nm. Figures are not on scale.

The technique of TEM is based on an electron beam that passes through the sample (thin sections of 60–80 nm of tissue or cultured cells) such that the beam will be absorbed and scattered by the sample, producing contrast that can be imaged (Winey et al., 2014) (Figure 4A and Figure 6). Using electrons as a source instead of light has the advantage that imaging can be performed at a much higher spatial resolution. The wavelength of an electron beam is much shorter than that of visible light and images with a resolution up to 2 nm can be obtained. Therefore, EM can be applied for high-resolution (ultra)structural analysis of whole tissues. Disadvantages are the limited sample depth the electrons can penetrate, imaging is done under vacuum conditions and there can be radiation damage. Therefore, microscope design, sample preparation, imaging and image processing must be carefully optimized. The quality of an image is mainly defined by three factors: contrast, resolution, and SNR [reviewed in (Franken et al., 2020)]. Staining of the sample with heavy metals is performed to increase the scattering events caused by the electrons when they interact with the sample, leading to a higher contrast of the image. Because the specimen chamber is under vacuum, samples need to be highly processed and are no longer in the natural state. Samples can be prepared by chemical fixation, dehydration, and staining with heavy metals which enhances contrast as mentioned above and protects somewhat against dehydration and radiation damage. Another way to preserve the tissue is cryo-fixation (cryo-EM); i.e. samples on a grid are frozen (either by plunging into a liquid cryogen or placing under high pressure, called plunge-frozen or high-pressure frozen respectively), using liquid ethane or ethane/propane to avoid the formation of ice crystals. Only thin samples (less than ≈10 µm) can be plunge-frozen, while specimens up to 300 µm can be high-pressure frozen, to achieve sufficient freezing of the sample, including the center (Dubochet et al., 1988). Cryo-EM can be performed without heavy metal staining and the contrast is then based on phase contrast only. The advantage is that it resembles the most native state and results in a high resolution. Thin samples are obtained by a process called ultramicrotomy, which can be done at room temperature or at sub-zero temperatures which can be challenging (Richter et al., 2007). In order to create thin sections from thicker samples, recent developments including cryo-EM of vitreous sections (CEMOVIS), or cryo-focused ion beam (FIB) milling can be used to create thinned areas within cryo-frozen specimens [reviewed in (Franken et al., 2020)]. Imaging of cryo-frozen samples require imaging in a cryogenic environment, which is technically demanding. High-pressure frozen samples can be stained by freeze-substitution, embedded in plastic resin, cut by ultramicrotomy and imaged at room temperature. More information about TEM of biological samples can be found in a book chapter (Mielańczyk Ł and Romuald, 2015).

EM made a major contribution to the visualization of intercellular structures by unraveling the adhesions in high detail. Ideally, the connection of the junctions to the cell’s cytoskeleton and membrane elements are imaged together to observe how they make an active junctional complex (see below). Additionally, individual proteins can be localized in context of the 3D volume.

Overall, researchers see that desmosomes are electron-dense and surrounded by a fuzzy area while adherens junctions are less electron-dense with the same intermembrane distance. For tight junctions however, there are different observations. In chemically fixed intestinal mucosa of the rat, tight junctions were seen as electron-dense apical membrane contacts forming a continuous belt-like attachment over a 200–500 nm distance by bringing the adjacent cell membranes in very close proximity (kissing points) (Figure 4A1) (Farquhar and Palade, 1963). Later, FFEM showed that tight junctions form interconnecting strand networks between cells that vary in number and morphology among different tissues. The frozen biological samples (with possible chemical fixation prior to freezing) are physically broken apart, typically fracturing at weak hydrophobic sites such as membranes, and a platinum-carbon replicate of the fracture plane is then analyzed by TEM, exposing the intercellular junctions between cells (Figure 4A3) (Chalcroft and Bullivant, 1970; Claude and Goodenough, 1973; Staehelin 1973; van Deurs and Koehler, 1979; Furuse 2010). FFEM has a limited spatial resolution due to the inherent property of metal atoms to crystallize. Recently Krystofiak et al. used a combination of amorphous carbon replicas with phase-contrast EM (Danev et al., 2014) to overcome this (Krystofiak et al., 2019). With this approach, they found that tight junction strands have an antiparallel double-stranded morphology.

When looking at the connection with the cytoskeleton, early observations using TEM of chemically fixed tissue section demonstrated that tight junction-associated actin filaments occur predominantly at sites of intercellular membrane apposition. These actin filaments are decorated by the actin binding region of myosin and appear to insert directly into the submembrane tight junction space (Madara and Pappenheimer, 1987). However, TEM of quick-freeze, deep-etch, rotary shadow replicas made it possible to distinguish between two different actin populations in epithelial cells (Höflinger 2014). Actin at the adherens junctions is organized as a ring and is composed of filaments that run parallel to the plasma membrane. This differs from the actin filaments found just beneath the tight junction membrane; these are organized as a meshwork of filaments (Hirokawa and Tilney, 1982; Hirokawa et al., 1983). Later, similar TEM-based techniques showed different features of the adherens junctions in a single layer of epithelial cells (chemically fixed and frozen). When sections were cut perpendicular to the cell membrane it revealed that adherens junctions have a 15–25 nm space between both membranes (Figure 4A2). With freeze-etching, it became clear that they are made up of a cytoplasmic macromolecular complex that consists of rod-like structures extending from the extracellular surface into the intercellular space, presumably catenin proteins of which the extracellular part may cant at about 60° with the plasma membrane (Miyaguchi 2000; Sluysmans et al., 2017). The standard TEM of chemical fixed cultured cells grown on a grid confirmed that the actin filaments run parallel to the adherens junctions (Buckley et al., 2014).

Cryo-EM of desmosomes in high-pressure frozen tissue sections showed that the desmosomal intercellular space has been variously reported as between 20 and 35 nm wide (Al-Amoudi et al., 2004). It is characterized by the presence of a dense midline with cross-bridges extending to the plasma membrane. When injecting the extracellular space of desmosomes with the electron-dense tracer lanthanum, researchers saw that the cross-bridges extending to the plasma membrane appear to alternate on opposite sides of the midline and that the desmosomal cadherin extracellular domains are ordered in all 3 planes (x, y, and z) (Rayns et al., 1969). So, the high resolution of EM and related techniques, including tracers and freeze-etching, allows researchers to distinguish the extracellular domains of cadherins in the intercellular junctions (Miyaguchi 2000; Al-Amoudi et al., 2004).

An evolved technique of rotary shadowing EM to reveal the surface topography of a sample is platinum replica EM (PREM). PREM is a specific type of EM where a cell on a glass coverslip is extracted or unroofed, followed by fixation and critical point drying. Then platinum is evaporated on a 3D sample at an angle during rotation of the sample after which carbon is reinforced on it (Heuser 1981). This reveals the topography and makes it an ideal technique for the structural analyses of the cytoskeleton (Svitkina and Borisy, 1998; Svitkina 2016). PREM achieves the high resolution typical for EM and the cytoskeletal structures are well preserved with visibility of single filaments, even if they are densely packed. Sample preparation for PREM is fast and efficient making it an inexpensive and accessible technique. However, only thin samples can be imaged as only the surface is made visible. The technique requires samples to be attached to glass surfaces which is not ideal to study membrane structures. Because of its informative value for cytoskeleton studies, PREM has been used and revealed the double-stranded morphology of the tight junction intermembrane fibrils (Krystofiak et al., 2019). In combination with light microscopy and immunolabeling for vascular endothelial cadherin with immunogold, it revealed the relationship between the parallel extensive branched actin network and the junctions between two endothelial cells (see Figure 4A4) (Svitkina 2017; Efimova and Svitkina, 2018). Specifically, the vascular endothelial cadherin colocalizes with the Arp2/3 complex of the F-actin network at different adherens junction types and is positioned at the interface between two oppositely oriented branched networks from adjacent cells. It is also proposed that the actin cytoskeleton at adherens junctions is a dynamic push-pull system (Efimova and Svitkina, 2018).

TEM allows imaging of intercellular junctions and ultrastructure of tissue or cultured cells at a very high (nanometer) resolution. Junctions in a cell layer are imaged perpendicular to the cell surface layer and with some techniques (like freeze-etching and PREM), structures just beneath the cell surface can be exposed and imaged. This is a suitable technique for visualizing the morphology of junctions, unraveling the relationship between the actin cytoskeleton and the intercellular complexes—with PREM it is even possible to see individual filaments in a 3D manner—and for measuring the intermembrane space between cells or see the extracellular domains of cadherin proteins. PREM requires some technical handling of the samples and optimization of the sample preparation.

### 3.2 3D Structural Information

When imaging in 2D, the spatial relationship between the optical axis of the microscope and the sample is important to interpret the way one looks at the junctions. When imaging samples in 3D, however, this is (partly) overcome by combining multiple 2D images, either from different angles—like electron tomography (ET) and soft X-ray tomography (SXT)—or from a stack of images that represent a voluminous sample (scanning EM, SEM). When reconstructing intercellular junctions, they can be seen from all sides in relation to the surrounding cells.

#### 3.2.1 Electron Tomography

In 2D it is difficult to interpret the complexity of the junctions; 3D imaging gives more accurate data. ET is an EM-derived technique and is based on a tilt series of 2D images acquired at different viewing angles, which are subsequently aligned and combined digitally into a 3D volume (see Figures 4B, 6). For an ideal reconstruction, tilt series contains projections from a ±60–70° rotation of the object to include projections from all possible directions [reviewed in (Ercius et al., 2015)]. This gives a 3D view through the depth of the specimen at a high resolution. Single-axis ET can cause imaging artifacts (known as the missing wedge problem) such as elongation of structures in the z-direction and loss of information in the x-direction (perpendicular to the tilt axis). Developments of dual-axis imaging partially overcome this problem (Guesdon et al., 2013). Sub-tomogram averaging can additionally be used to reduce the imaging artifacts and decrease the SNR. By making several images at different angles the resolution of ET is higher compared to EM, particularly in z, and especially when tomograms are acquired from viewing at two different axes. Cryo-ET is often used to preserve the sample in its native state. In combination with sub-tomogram averaging, it results in an atomic resolution (5–10 Å) and allows for single particle analysis (Nakane et al., 2020; Yip et al., 2020; Zhang et al., 2020), but it has not yet been reached with imaging of biological cells or tissue. This technique provides molecular resolution 3D images of cellular landscapes and can visualize the junction from different angles in the tissue, not just perpendicular to the cell membrane. ET is, as TEM, restricted in volume because the sample must be thin sections (limited to about 300 nm) to render them electron transparent. Thicker samples can be imaged with the of more sophisticated approaches and instrumentation (Lučič et al., 2013).

Freeze-substituted ET revealed that desmosomes are more loosely arranged (He et al., 2003) (Figure 4B1) compared to those seen using CEMOVIS from a ca. 150 μm thick fully vitrified biopsy (Al-Amoudi et al., 2004). However, using CEMOVIS data results differ from what has been seen before. It has to be noted that those types of samples are typically larger by 15% or more compared to fixed, dehydrated specimens [reviewed in (Al-Amoudi et al., 2004)]. Cryo-ET experiments combined with sub-tomogram averaging and atomistic molecular dynamics simulations demonstrated that cryo-ET is a powerful method for high-resolution imaging. It was able to reveal discrete groups of cadherin molecules with different plausible cadherin arrangements, and their *cis* and *trans* interactions in desmosomes (Sikora et al., 2020).

Using ET, researchers made a 3D reconstruction of an intercalated disc, a component of the connection between murine cardiac muscle cells where mainly gap junctions, desmosomes and a mixed type of junction (area compositae) are located (Borrmann et al., 2006; Franke et al., 2006). This revealed the pattern and the presence of desmosomes and mixed junctions in 3D (Leo-Macías et al., 2015).

ET can obtain higher resolution than traditional TEM and also has the advantage of imaging samples in 3D. ET can be used to study the filament arrangement within the junctions and unravel the binding interactions (*cis* or *trans*) between substructures of the intercellular junctions. It can also be used as a quantitative tool of the intermembrane distances in 3D. The sample preparations, technical experience and microscope availability are similar to that of TEM.

#### 3.2.2 Volume-Electron Microscopy

Volume scanning EM (volume-SEM) offers the opportunity to image larger volumes with a greater sample depth compared to ET. The technique is based on altering between scanning the tissue surface with a focused electron beam followed by cutting off a section of the *en-bloc* tissue (Figure 4B and Figure 6). This generates a large stack of 2D images on which segmentation is performed to render 3D reconstructions of the cell with its subcellular structures. Cutting the section is done by either a diamond knife (serial block-face (SBF)-SEM) or an ion beam (focused ion beam (FIB)-SEM), enabling the cutting of sections with a thickness of 25–50 nm and 5–20 nm respectively. The resulting high-resolution voxel sizes are up to 10 × 10 × 25 nm^3^ for SBF-SEM (Wanner et al., 2016) and 5 × 5 × 5 nm^3^ for FIB-SEM (Knott et al., 2011; Boergens and Denk, 2013). The maximum sample width for SBF-SEM is around 1 mm, while FIB-SEM is limited to a much smaller imaging field of 20–100 µm width (Titze and Genoud, 2016). The volume SEM can image depends on the available imaging time; the more volume you image the longer it takes. The sample preparation needs optimization per sample type, and often includes heavy metal staining for contrast and embedding in plastic resin. On top, coating of the sample with a thin layer of conductive material (i.e., gold) is performed to reduce the charging effects. Both SBF-SEM and FIB-SEM have been used to study the intercalated disc between cardiac muscle cells in 3D, along with the desmosomes and gap junctions of murine cardiac tissue (Figure 4B2) (Vanslembrouck et al., 2018; Vanslembrouck et al., 2020). The heavily folded intercalated disc is clearly visible, where two different regions can be distinguished with a different pattern of junctions on them. Moreover, FIB-SEM has revealed that in diseased mice, the intercellular junctions behave differently in a region-specific manner.

FIB-SEM is a useful, modern technique which is shown by the study that looked at the detailed location of the SARS-CoV2 virus particles in human lung epithelial cells to accurately quantify virus density and surface curvatures. Results of the FIB-SEM imaging and 3D reconstructions of interactions between the SARS-CoV-2 virus and the cell revealed a tight junction-mediated contact between adjacent cells with a dramatic surface viral density difference on either side of the tight junction (Figure 4B3) (Baena et al., 2021).

Volume-SEM is a great tool to visualize intercellular junctions and the connecting membrane in a voluminous and quantitative way at high resolution in 3D. It can be used to image the relationship between different junctions in a volume, their localization on the membrane surface, and the relationship to the other substructures of the cell. Immunogold labeling of tissue imaged by SEM has been done previously, but not yet for intercellular junctions. This could however, with sample optimization, be a very helpful approach to image individual proteins in their 3D imaged junctions (Goldberg and Fišerová, 2016). However, the main disadvantage of volume-SEM is the cost and technical handling of the machine, together with a need for proper optimization of the sample preparation. Even though there is no broad availability of this technique, the microscopes are often part of a service facility that helps the researcher with all the technical aspects, sample preparation and data interpretation.

#### 3.2.3 Soft X-Ray Tomography

X-rays can be used as an illumination light source while a wide range in resolution can be achieved using different energies with corresponding X-ray optics (Rawson et al., 2020); i.e. microtomography with a spatial resolution from 1 µm up to 100 µm and nano-imaging with a resolution down to 10 nm voxel size. The principle of X-ray tomography is based on taking projection images using X-rays from different angles around a sample. This is followed by reconstruction and segmentation to obtain 3D volumes and volumetric quantifications (Bradley and Withers, 2016; Guo and Larabell, 2019; Töpperwien et al., 2020) (Figure 6). Using X-rays generated at a synchrotron, images with a nanometer resolution (50 nm or better) can be generated. Because of the destructive nature of X-rays, samples are plunge frozen and thus imaged as close to the native state as possible due to cryofixation of the samples (Krenkel et al., 2015). SXT images the sample in the water window (Weinhardt et al., 2019), meaning that X-rays are absorbed by carbon and nitrogen in biological tissue more than by oxygen in water, resulting in grayscale data that can be used for quantitative analysis. However, limited penetration of soft X-rays results in a limited sample thickness of 10–20 µm (Carzaniga et al., 2014; Duke et al., 2014).

SXT has mainly been used to image adherent cells cultured on 2D surfaces or in cell suspension (Elgass et al., 2015; Harkiolaki et al., 2018). The resolution of this technique allows researchers to study the adhesion complexes between cells, as the plasma membrane is clearly visible in several studies (Peter Szekeres et al., 2020). However, the focus of most of these projects is the nucleus, lipid droplets and intracellular structures. Often, 3D reconstructions of single cells are the main goal with SXT, but imaging of multiple cells has been done (Loo Jr et al., 2001; Loconte et al., 2021) and efforts are evolving to image cell-cell connections between adherent cells (own unpublished results). For example, researchers saw multinucleated cells in SARS-CoV-2 infected embryonic kidney cells which could have points of interaction made visible with SXT, as seen for desmosomes in the FIB-SEM images from SARS-CoV-2 infected human lung cells (Baena et al., 2021; Loconte et al., 2021).

SXT allows the researchers to image intercellular junctions at nanometer resolution in the near-native state, and it is possible to reconstruct the ultrastructure of the cell in 3D. Even though imaging of samples is at cryogenic temperatures, only thinner samples (max of 10 microns) can be efficiently frozen as SXT is up to date limited to plunge freezing. To our knowledge, it has not been used yet to image desmosomes, adherens junctions or tight junctions. However, with some sample preparation optimization, we are convinced that the resolution of the technique allows the researcher to image these substructures. Correlation with light microscopy is also an option (Ekman et al., 2019), which makes it a good technique for studying cell-cell adhesion complexes.

### 3.3 Compositional Information

#### 3.3.1 Immuno-Electron Microscopy

In parallel with fluorescence imaging (see part 2.2), immuno-labeling with a certain tag/antibody can also be performed when imaging with electrons. Immuno-EM is a very useful technique to localize individual proteins at a nanometer resolution. It has the advantage that the ultrastructure of the junctions is still visible at the same time and in a single image. Immuno-EM has, however, limited molecular specificity, meaning that the antigen/antibody binding must be strong and specific in which proper controls are highly desirable to eliminate non-specific binding. Immunolabeling in TEM and SEM is a widely used technique in which different sizes of gold nanoparticles have been used to label different proteins resulting in multi-“colored” images (Koster and Klumperman, 2003; Straub et al., 2011; Boykins et al., 2016; Goldberg and Fišerová, 2016; Jones 2016). It can be, however, technically challenging and proper sample preparation is required (Griffiths and Lucocq, 2014). Immunolabeling can generally be done in two ways depending on the embedding process: pre- and post-embedding. During pre-embedding, the labeling is carried out on micrometer-thick cryostat sections of frozen tissues or chemically fixed tissue followed by embedding in resin followed by sectioning. Pre-embedding has the advantage of strong immunolabeling and preserving the antigenicity but is poor in preserving ultrastructural details. Post-embedding requires the antibody to bind on the surface of the ultrathin section and requires thawing of frozen sections with cryoprotection and hydrophilic resins, like the Tokuyasu method (Tokuyasu 1973). This technique is a better choice to preserve the ultrastructure. Recent developments exist that combine the benefits of both techniques (Jones 2016; Kim et al., 2021).

Immuno-EM helped already in the early ages of junctional research in numerous studies to identify the individual proteins located at the intercellular junctions as well as their distance to both the transmembrane and peri-junctional part of the cell (Stevenson et al., 1986; Jesaitis and Goodenough, 1994). For example, immuno-EM labeling was crucial for the identification of occludin and claudin protein at freeze-fractured tight junctions (Furuse et al., 1994; Furuse et al., 1998).

In a more recent study that investigated endocytosis in epithelial cells (enterocytes of mice), antibodies against occludin and caveolin-1 with a different size of gold particle were used. They observed that both proteins were present at the apical junction complex, with occludin at the tight junction while caveolin-1 was detected at the adherens junction and, to a lesser extent, at the tight junction. They also noticed that the co-localization pattern is disturbed after pharmacological intervention resulting in loss of the tight junction barrier (Figure 3D) (Marchiando et al., 2010). Colocalization immuno-EM was also a crucial method to study the area compositae at the intercalated disc of cardiac muscle cells. Using this technique, researchers identified the mixed type of junction where proteins previously belonging to one type of junction are seen together, such as plakoglobin (Franke et al., 2006).

An alternative method to localize junctional proteins in EM by gold-conjugated antibodies, is the use of genetic tags that generate EM contrast on a specific protein or subcellular structure of interest. This has the advantages that it does not require permeabilizing treatments and is a robust method. Examples are APEX2 and mini-SOG. Specifically, APEX (enhanced ascorbate peroxidase) is an enzyme that catalyzes the H_2_O_2_-dependent polymerization of DAB into an insoluble polymer. This DAB polymer is osmiophilic, which becomes EM-visible after treatment with OsO_4_. APEX does not need any light, has minimal diffusion of the reaction product, works in presence of glutaraldehyde fixation and yields excellent preservation of the ultrastructure (Martell et al., 2017). In a recent study, researchers used APEX2 (a better and more recently developed APEX mutant) with EM imaging in combination with quantitative proximity proteomics. It revealed the molecular and spatial organization of the apical junction complex (mix of tight and adherens proteins) and the junction-associated polarity network in fully polarized epithelial cells. The researchers showed using APEX labelling that the Crumbs complex (module) localizes to a distinct cortical region just apical of tight junctions (Tan et al., 2020). MiniSOG contains 106 amino acids, less than half the size of green fluorescence protein. Illumination of miniSOG generates sufficient singlet oxygen to locally catalyze the polymerization of diaminobenzidine into an osmiophilic reaction product resolvable by EM (Shu et al., 2011).

Immuno-EM has the main advantage that it can label individual proteins and see the ultrastructure of the sample at a nanometer resolution. Even though it is a destructive technique that needs sample optimization, it is accessible to many researchers. Immuno-EM is, together with super-resolution microscopy, often used to confirm protein (co)localization and interactions within and around the intercellular junction of interest. The technique that researchers use depends on the availability of the microscope.

## 4 Combining Both Structure and Composition With Correlative Imaging

A rising field is EM combined with fluorescent techniques, called correlative light-EM (CLEM) in which the same field of view is imaged by both modalities. The fluorescent modalities can be widefield, confocal or super-resolution microscopy. This enables the analysis of molecular-scale resolution in a (sub) cellular/ultrastructural context. In the past decade, CLEM had a boost because of the development and optimizations of better probes, sample preparation, super-resolution fluorescence microscopy, and data handling. This causes a better match in terms of resolution between the two modalities but also the ability to image larger volumes (Guerin and Lippens, 2021).

Overall, there are two ways to perform CLEM. Live cell fluorescent imaging of the samples can be performed first followed by sample preparation for and imaging with EM. The second approach is performing light microscopy and EM on the exact same sample, once it has been prepared for EM. This can be applied to embedded samples in resin (with a pre-or post-embedding approach) or cryo-samples [reviewed in (de Boer et al., 2015)]. Good sample preparation in CLEM is very important as routine fluorescence procedures and EM preparation are often mutually exclusive because either fluorescence is lost, or the ultrastructure is destroyed.

For CLEM experiments, samples are transferred between fluorescence microscopy and EM modalities in which matching the observed areas and identification of the region of interest with both is crucial [reviewed in (de Boer et al., 2015)]. Used methods are for instance grids that enable coarse alignment of a sample, commercial sample holders with navigation markers recognized by microscope software and branding optical marks in the sample with laser irradiation. For high-precision overlay, fiducial markers, that can be identified by both light microscopy and EM are used and can be an integrated part of the sample or added below the sample.

Next to fluorophores, quantum dots are used and can be visualized both in fluorescent settings and have a dense metal core making it visible with EM (Clarke and Royle, 2018; Rae et al., 2021).

Cryo-SIM/FIB-SEM correlation of cell-cell adhesions in mouse cerebral granule neurons showed that the adhesion molecule JAM-C between two labeled somas was not uniform but formed a web-like structure at their shared membrane contact zone (Hoffman et al., 2020). They also detected that the adhesion does not comprise the whole contact area between two cells, as expected because of the mechanical tension on adhesion complexes. On top, Drebrin (a cytoplasmic actin-microtubule cross-linker protein) is enriched in the regions adjacent to JAM-C which contrasts the laminar stacking of adhesion-associated cytoskeletal adaptor proteins found in focal or cadherin-based adhesion on glass (Kanchanawong et al., 2010; Bertocchi et al., 2017).

## 5 How to Choose the Right Imaging Technique for Your Intercellular Junction Research?

In the previous sections, many techniques showed their contribution in unraveling the protein composition or the connection between intercellular junctions and the cell’s cytoskeleton. Next to the structure and composition of the junctions, many microscopy techniques can also be used to discover their activity, including the dynamics and functions which has not been reviewed here. Visualizing cell-cell adhesions can be particularly challenging because of their membrane localization, molecular complexity, and small size. All discussed techniques in this review have their own specific (dis) advantages and possibilities and must be taken into consideration before starting an experiment.

### 5.1 Sample Preparation and Sample Type

One of the main considerations is the sample preparation and the type of sample you are dealing with. There is the possibility to perform *in vitro*, *in vivo,* and *ex vivo* imaging. Some techniques do not allow for *in vivo* imaging, for instance due to the destructive nature of the microscope, while all super-resolution techniques do allow live imaging. Intravital microscopy for instance provides imaging of cellular events in its native tissue environment as well as in a real-time setting. It has several compelling advantages to study intercellular junctions. For example, researchers visualized E-cadherin labeled cell-cell junctions in mouse pancreas using intravital microscopy combined with multiphoton imaging (Erami et al., 2016). However, imaging in live animals has many technical challenges while it also requires access to high-resolution microscopy techniques (reviewed in (Choi et al., 2015; Hickey et al., 2022). Smaller mammalian species might be considered, including larval zebrafish, because of their high optical transparency that enables direct visual access to biological processes (Ahrens et al., 2012; Høgset et al., 2020). Next to imaging in live animals, freshly excised organs can be studied, followed by optional processing (fixation, freezing or staining) a process termed *ex vivo* imaging. This processing can alter the native state of the tissue and the morphology, as well as the interpretation of functional studies. However, it is still one of the preferred methods for imaging intercellular junctions (see below) and possible with all discussed imaging techniques. Finally, *in vitro* imaging (imaging of cell cultures; imaging can be performed with all discussed techniques) has many advantages, including reduced cost, higher throughput, ease to manipulate and reduced need for animals. The most significant drawback is that cell culture models may fail to replicate *in vivo* biology since they lack the complex network of interactions or intercellular signaling, which occurs in tissues, organs or whole organisms. Recent advances do include co-cultures of multiple cell types and researchers are increasingly using 3D cell models to recreate organized, miniaturized versions of organs to study diseases (Martin 2020). All three types of imaging ways have their advantages and disadvantages as well as technical difficulties and necessary equipment (reviewed in (Hickey et al., 2022)).

*In vivo* imaging enables the study of dynamic cell behavior but is not possible with techniques that use electrons or X-rays. Another variable is the temporal resolution when imaging with confocal and super-resolution microscopy. Some techniques, like SIM, allow for a very fast imaging speed (sub-seconds) while others are much slower (including SMLM, seconds to minutes and STED, 10 ms to minutes). In living cells, direct *in vivo* visualization of protein-protein interactions is also possible with a technique called bimolecular fluorescence complementation (BiFC) (Hudry et al., 2011). For instance, this technique helped researchers to unravel the role that the tight junction claudin-2 plays in the entry of the reovirus into the cell (Zhu et al., 2021). Examples of which super-resolution techniques that can be used for tight junction and adherens junction visualization in live-cell or in fixed samples are reviewed in (Gonschior et al., 2020).

*In vitro* imaging of cultured cells is a standard approach in biological research. However, when fully polarized epithelial cells, in monolayer, are grown on a 2D support (such as a glass coverslip), the intercellular junctions are located 10–20 microns away from the growth substrate and are thus not suitable to be imaged with super-resolution techniques like SMLM (as explained above). The principle and important considerations for tight and adherens junction imaging are reviewed (Gonschior et al., 2020). Other approaches, including cells grown on a filter membrane followed by mounting on a coverslip [as described above (Maraspini et al., 2019)] can overcome this orientational problem during imaging. This still requires the cell-cell junctions to be imaged along the optical axis, which provides only limited information on their organization along the lateral membrane. The axial resolution of most light-based techniques is not enough when imaging deeper in the cells. Next to growing cells in 3D [like organoids (Martin 2020; Hickey et al., 2022)], imaging epithelial or endothelial tissue sections is another good approach. Tissue sections have the major advantage of having preserved complex interactions among cells and their microenvironment and therefore cells kept their cell polarity in the hierarchical architecture of the tissue. It also allows the researchers to image cell junctions in a side view (x/y) when cut at the right angle. With 3D imaging techniques (such as ET, SXT and volume-SEM), the junctions can be seen from different angles, reconstructed as one volume among cells and analyzed from different orientations.

Not only the way of sample imaging, but also the state in which it is imaged is a key step in the decision-making process. Some imaging techniques require fixation because of their destructive technique, for example when imaging with electrons and X-rays (Popescu et al., 2016). New developments are emerging to overcome this issue (Elphick et al., 2021). Chemical fixation sometimes followed with the embedding of the sample in plastic for EM processing, is a widely used technique but can affect the structure of the sample due to dehydration. To make sure the ultrastructure is preserved, cryo-freezing has become a more popular fixation method in recent years; it gives excellent preservation of the material in a near-native state compared to chemical fixation (Dahl and Staehelin, 1989; Shimoni and Müller, 1998). Sectioning cryogenic material (cryo-ultramicrotomy) for the preparation of ultrathin sections, as necessary for TEM, involves sophisticated tools and is technically demanding (Studer et al., 2008; Glaeser et al., 2016). Most fluorescent imaging techniques, either confocal or super-resolution, allow for imaging on living cells/samples within a controlled environment and the possibility of time-lapse imaging for days. However, possible phototoxic events after long illumination, as seen in super-resolution techniques like STED and SIM, need to be taken into consideration [reviewed and solutions in (Tosheva et al., 2020; Hickey et al., 2022)].

In some cases, it is very difficult to image the intercellular junctions in their native localization for several reasons. Therefore, researchers have engineered other techniques that allow them to image individual proteins/complexes at a high resolution. For instance, the cryo-EM image of isolated desmosomal fractions has been used to produce a molecular model of desmosomes (Sikora et al., 2020). Even though this has helped researchers to understand some biological questions, the physiological relevance of this type of set-up can be discussed.

### 5.2 Orientation of Imaging

Different techniques have different imaging depths, as discussed above (Table 1 and 2). Intercellular junctions are located at the lateral membrane perpendicular to the optical axis of most techniques (Figures 5, 6). Depending on the cell type studied the junctions are hundreds of nanometers to tens of micrometers away from the coverslip (Figure 1), thus they require an imaging technique with a certain illumination depth.

Researchers have developed many adjustments to confocal and super-resolution techniques to meet these requirements. However, the higher the lateral (xy) resolution, the more superficial the imaging must be. For instance, a technique with a very high lateral (xy) resolution is TIRF microscopy. The excitation light in TIRF is shined at a very high angle, causing it to internally reflect and no out-of-focus light is generated as only a very thin (∼0.2 um) layer adjacent to the coverslip is excited, with a high SNR as a result (Fish 2009; Mattheyses et al., 2010). TIRF is often used as a setup during SMLM imaging which gives superb lateral (xy) resolution. However, only a limited field of view can be imaged and junctions within the tissue cannot be resolved with this technique (Tokunaga et al., 2008; Gerb and Jacob, 2011). Moreover, combination with other super-resolution methods with more specialized illumination geometries such as two-photon or light-sheet in addition to tissue-clearing and adaptive-optics based corrections could be promising to unravel the complex nature of junctions [as reviewed in (Bond et al., 2022)]. However, this has not been performed much for tight junctions, adherens junctions or desmosomes. Tissue clearing gives the opportunity to image with reduced light scattering and increased imaging depth with a range of microscopic and tomographic methodologies of larger and thicker samples (Almagro et al., 2021). Developments in the optics aspect of microscopy enabled researchers to image, with SIM, samples thicker than a single cell (Lin et al., 2021). This could be promising to image junctions in tissues instead of cell cultures with super-resolution microscopy.

If one wants to image deeper into the tissue and look at larger volumes, 3D imaging techniques using X-rays and electrons are ideal. Using volume-SEM, ET and SXT allow researchers to image deep into tissue, in intact organisms and to reconstruct the intercellular junctions in 3D. However, it is limited to fixed samples (chemically or cryo-frozen; as mentioned above).

### 5.3 Labeling and Controls

During imaging, researchers want to image the correct protein(s) and need to be sure that what they see is what they intended to see. Labels/probes need to bind the protein of interest correctly and these can be directly visualized or followed by targeting of an imaging label (in case of fluorescence microscopy) or gold particle (in case of EM). Therefore, applying appropriate controls when setting up an experiment is highly desirable [discussed in (Hickey et al., 2022)]. The easiest are techniques without the need for labelling to see subcellular structures, which include SXT and cryo-EM. The downside is that the distinction of the cellular components relies on the expertise of experienced scientists.

Antibodies are the most widely used probes, they are highly specific to their targets and there is a large selection available. However, they take up quite some space (15 nm in length) and their dual binding capability can introduce significant artifacts in super-resolution imaging [reviewed in (Fornasiero and Opazo, 2015)]. To reduce the size, antibody fragments or single-chain antibodies (nanobodies) were developed. As epitopes must be labeled at high density for accurate super-resolution imaging, the fluorophores must be as close as possible to their targets and nanobodies are perfect for this. However, one must make sure all endogenous proteins are imaged as antibodies have been known to miss some when detecting GFP-tagged proteins. Other options are aptamers, single-stranded DNA or RNA oligonucleotides that are selected *in vitro* for the desired targets, showing improved precision obtained in STED and SMLM when compared to antibody staining (Opazo et al., 2012). More small molecules and scaffolds are produced to make single localization imaging possible (Fornasiero and Opazo, 2015).

### 5.4 Post-Processing

Once images are taken, the data must be processed, stored and analyzed. This can get very complex, time- and computer-demanding depending on the technique used. 3D acquired data (from ET, SXT and volume-SEM) needs some post-processing, including corrections, stack alignments and sub-tomogram averaging, before analysis can be performed (Schur 2019; Pyle and Zanetti, 2021). EM and SXT data analysis is often carried out by specialists with experience in the identification and interpretation of biological features in the complex grayscale world of this type of imaging. Manual analysis is labor-intensive and slow. Automation with the use of machine learning is needed to accelerate the speed and efficiency of segmentations and structural analysis (Peddie and Collinson, 2014; Ekman et al., 2020; Lösel et al., 2020). Also, methods for good data storage and computing are evolving (Baldwin et al., 2018; Vescovi et al., 2020).

Evolution to nanometer resolution microscopy has been extremely fast in the last decade. The increased resolution imposes stringent conditions on data analysis and researchers must ensure that photophysical properties of the probes, the labeling, and the imaging strategies are correct and do not lead to misinterpretation of the data. New methods have been developed for proper analysis and quantification of imaged data by super-resolution techniques (Durisic et al., 2014). For the super-resolution techniques, SMLM data analysis is quite computational demanding, while SIM is less and STED is fairly straightforward (Bond et al., 2022).

### 5.5 Using More Than One Technique to Answer the Research Question

Combining different, yet complementary, imaging modalities offers the advantage of obtaining images originating from distinct contrast mechanisms. This is of great help as it will provide information with different resolution ranges. For example, EM and confocal imaging unraveled a close relationship of tight junction and adherens junctions proteins, with claudin as a central protein (Nunes et al., 2006). A combination of SIM with PLA showed that clusters within one adherens junction are united by the same actin filament bundle, but this interaction is not uniform among all adherens junctions (Indra et al., 2013). The same researchers recently showed that actin bundles at punctate adherens junctions consist of two distinct regions—a stable stalk and a highly dynamic cadherin-interacting tip (Indra et al., 2020). The F-actin turnover differs in the structures with a faster turnover in the tip but is dependent on the cadherin clusters. This confirms the bidirectional coupling of the cytoskeleton with the cadherin-catenin complex of the adherens junctions to coordinate the actin dynamics between neighboring cells. This study is consistent with the findings of Efimova et al. using PREM to study the actin bundles at the endothelial adherens junctions, see above under 2.1 (Efimova and Svitkina, 2018). This illustrates that new developments in microscopy and the use of multiple imaging techniques give more insights and information about the complex organization of the adhesion complexes. Moreover, the combination of imaging techniques with biochemical assays helps the researchers to understand the function of the junctional (sub) structures (Esposito and Venkitaraman, 2019). Combining techniques can also be used for practical implications during imaging. For instance, to find cells of interest at a low resolution followed by imaging at a high resolution (Guérin et al., 2019).

## 6 Conclusion

A broad range of techniques are available to image either the (ultra) structure and composition of the different intercellular junctions between endothelial and epithelial cells. Each method with its own possibilities and probable pitfalls (Table 1 and 2). With electrons and X-rays, nanometer resolutions can be achieved in 3D, unfortunately, it remains destructive and needs fixed samples. And even though it sometimes lacks sufficient imaging depth for studying intercellular junctions, super-resolution imaging gave a big boost to the research field. Overall, we can conclude that with the emerging new techniques, more powerful instruments and proper expertise, the future looks bright for revealing the remaining secrets of complex cell-cell, intercellular structures unresolved in many cell- and tissue types.

## Abbreviations

CEMOVIS Cryo-electron microscopy of vitreous sections; CLEM Correlative light electron microscopy; Dsg Desmoglein; dSTORM Direct stochastic optical reconstruction microscopy; EM Electron microscopy; ET Electron tomography; F-actin Filamentous actin; FFEM Freeze fracture electron microscopy; FIB Focused ion beam; FIB-SEM Focused ion beam scanning electron microscopy; FRET Forster resonance energy transfer; iPALM Interferometric photoactivated localization microscopy; JAM Junctional adhesion molecules; MinFlux Minimal photon flux imaging; PAINT Point accumulation imaging in nanoscale topography; PALM Photoactivated localization microscopy; PKP Plakophilin; PLA Proximity ligation assay; PREM Platinum replica electron microscopy; SBF-SEM Scanning block face scanning electron microscopy; SEM Scanning electron microscopy SIM Structured illumination microscopy; SMLM Single molecule localization microscopy; SNR Signal to noise ratio; STED Stimulated emission depletion; STORM Stochastic optical reconstruction microscopy; SXT Soft X-ray tomography; TEM Transmission electron microscopy; TIRF Total internal reflection fluorescence; ZO Zona-occludens.
